# Association between continuity of care and subsequent diagnosis of multimorbidity in Ontario, Canada from 2001–2015: A retrospective cohort study

**DOI:** 10.1371/journal.pone.0245193

**Published:** 2021-03-11

**Authors:** Edward Chau, Laura C. Rosella, Luke Mondor, Walter P. Wodchis

**Affiliations:** 1 Institute of Health Policy, Management, and Evaluation, University of Toronto, Toronto, Canada; 2 Dalla Lana School of Public Health, University of Toronto, Toronto, Canada; 3 Institute for Better Health, Trillium Health Partners, Mississauga, Canada; 4 ICES, Toronto, Canada; Universidade Federal de Pelotas, BRAZIL

## Abstract

**Background:**

Continuity of care is a well-recognized principle of the primary care discipline owing to its medical, interpersonal, and cost-saving benefits. Relationship continuity or the ongoing therapeutic relationship between a patient and their physician is a particularly desirable goal, but its role in preventing the accumulation of chronic conditions diagnoses in individuals is unknown. The objective of this study was to investigate the effect of continuity of care with physicians on the rate of incident multimorbidity diagnoses in patients with existing conditions.

**Methods:**

This was a population-based, retrospective cohort study from 2001 to 2015 that focused on patients aged 18 to 105 years with at least one chronic condition (n = 166,665). Our primary exposure was relationship continuity of care with general practitioners and specialists measured using the Bice-Boxerman Continuity of Care Index (COCI). COCI was specified as a time-dependent exposure prior to the observation period. Our outcomes of interest were the time to diagnosis of a second, third, and fourth chronic condition estimated using cause-specific hazard regressions accounting for death as a competing risk.

**Findings:**

We observed that patients with a single chronic condition and high continuity of care (>0.50) were diagnosed with a second chronic condition or multimorbidity at an 8% lower rate compared to individuals with low continuity (cause-specific hazard ratio (HR) 0.92 (95% Confidence Interval 0.90–0.93; *p*<0.0001) after adjusting for age, sex, income, place of residence, primary care enrolment, and the annual number of physician visits. Continuity remained protective as the degree of multimorbidity increased. Among patients with two conditions, the risk of diagnosis of a third chronic condition was also 8% lower for individuals with high continuity (HR 0.92; CI 0.90–0.94; *p*<0.0001). Patients with three conditions and high continuity had a 9% lower risk of diagnosis with a fourth condition (HR 0.91; CI 0.89–0.93; *p*<0.0001).

**Conclusions:**

Continuity of care is a potentially modifiable health system factor that reduces the rate at which diagnoses of chronic conditions are made over time in patients with multimorbidity. Additional research is needed to explain the underlying mechanisms through which continuity is related to a protective effect and the clinical sequalae.

## Introduction

A notable challenge for healthcare systems arising from the aging of populations and improvements in life expectancy is the increase in the number of patients with chronic conditions. The proportion of patients that are now living with multimorbidity or more than one condition at the same time in particular has risen considerably. This is an issue in Ontario, Canada where estimates indicate that nearly a quarter of the population is multimorbid, with 83% of the elderly having been diagnosed with multiple chronic conditions [1]. From the perspective of policymakers, multimorbidity needs to be addressed urgently because it results in substantial resource use leading to concerns about the long-term financial sustainability of health systems. It has been shown that the more than two-thirds of health care expenditures are attributable to to the population with 2 or more chronic conditions [2]. Importantly, multimorbidity causes marked deteriorations in health as the development of more conditions raises the risk of mortality, functional disability, polypharmacy, and poor quality of life [3–7].

Recently, there has been considerable interest in the role that continuity of care has in affecting certain health outcomes [8–12]. Continuity is an aspect of the care delivery process that reflects the patient experience of care over time, with three inter-related elements: informational, management, and relationship [13]. Informational continuity is recognized as the care that patients receive that is informed by their past medical history, management continuity is the coordination and integration of care that follows a shared care plan, and relationship continuity is the presence of an ongoing therapeutic relationship between patients and their providers [13,14]. While all types of continuity are valuable in their own right, relationship (or clinician) continuity resonates strongly with both patients and general practitioners [10]. Empirical studies have shown that the risk of all-cause mortality, emergency department visits, and hospitalizations is lower when there is high relationship continuity in physician care [8,15]. High relationship continuity has a similar effect on hospitalization risk as having one less condition amongst individuals with multimorbidity [9]. Enhanced continuity also protects against duplicated medications and adverse interactions between drugs, which is a prevalent issue among multimorbidity patients [11,12,16,17].

Given the rising incidence of multimorbidity and the consequences that are associated with the accumulation of chronic conditions, it is worthwhile exploring whether relationship continuity also affects the development of multimorbidity itself and to what extent as this may guide efforts to prevent its onset [18,19]. Most prior studies to date have concentrated on the sociodemographic and behavioural determinants of multimorbidity [20–23]. However, higher continuity could be associated with better health by improving patient health behaviours and initiate the creation of more consolidated treatment regimens. This would reduce the onset of worsening health and additional conditions. Existing studies of the association between continuity and the accumulation of disease are cross-sectional in nature and considered continuity as an outcome rather than an exposure [24,25]. While having multiple conditions can appropriately result in more physicians being involved thereby risking discontinuity of care, continuity itself may be protective against future conditions by supporting self-management and having a strong trusting relationship leading to increased treatment adherence [26–28]. The objective of this study was therefore to investigate the effect of relationship continuity of care, hereafter referred to as continuity, on the rate of developing developing new chronic conditions in patients with at least one chronic condition. Additionally, we assess the robustness of this association by examining whether continuity affects the rate of becoming diagnosed with a consecutive conditions in patients with a higher degree of multimorbidity.

## Methods

### Study design and setting

We performed a population-based, retrospective cohort study of patients with at least one previously diagnosed chronic condition in Ontario, Canada’s largest province (population of 13 789 600 in 2015). Provincial residents receive coverage for all medically necessary hospital and physician services through the publicly funded health insurance program known as the Ontario Health Insurance Plan (OHIP). All publicly paid services are recorded in health administrative databases maintained by prescribed entitites. This study used databases that were linked deterministically using unique encoded identifiers and analyzed at ICES, a not-for-profit entity that houses administrative databases and leverages them towards population-based health services research.

### Cohort

The study population included all Ontario residents aged 18 to 105 years who were diagnosed with osteo- and other arthritis, osteoporosis, renal failure, cardiac arrhythmia, coronary artery disease, non-psychotic mood and anxiety disorders, other mental health conditions (including schizophrenia, delusions, and other psychoses, personality disorders, and substance abuse), dementia, rheumatoid arthritis, chronic obstructive pulmonary disease, congestive heart failure, acute myocardial infarction, asthma, hypertension, diabetes, stroke, or any cancer as their first chronic condition during the accrual period of 1 April 2001 and 31 March 2003. The follow-up period was 12 years from 1 April 2003 to 31 March 2015. These conditions were selected based on their population and economic burden in Ontario and elsewhere [29–32]. Where available, we applied algorithms that were previously validated in Ontario health administrative databases against randomly selected primary care charts [33–40]. These algorithms were based on the presence of a relevant International Classification of Disease code in the OHIP database and hospitalization data from the Canadian Institute for Health Information (CIHI) Discharge Abstract Database (S1 Table). Ascertainment of dementia also considered claims for dispensed cholinesterase inhibitors in the Ontario Drug Benefit Claims Database as dementia is the only indication for these drugs [40]. In the absence of validated algorithms for the remaining nine conditions, we used the algorithm of one inpatient hospital diagnostic code or two or more outpatient physician billing codes in two years due to its consistency with the validated algorithms and previous population-based analyses of multimorbidity in Ontario [1,6,9,41].

**Fig 1:**
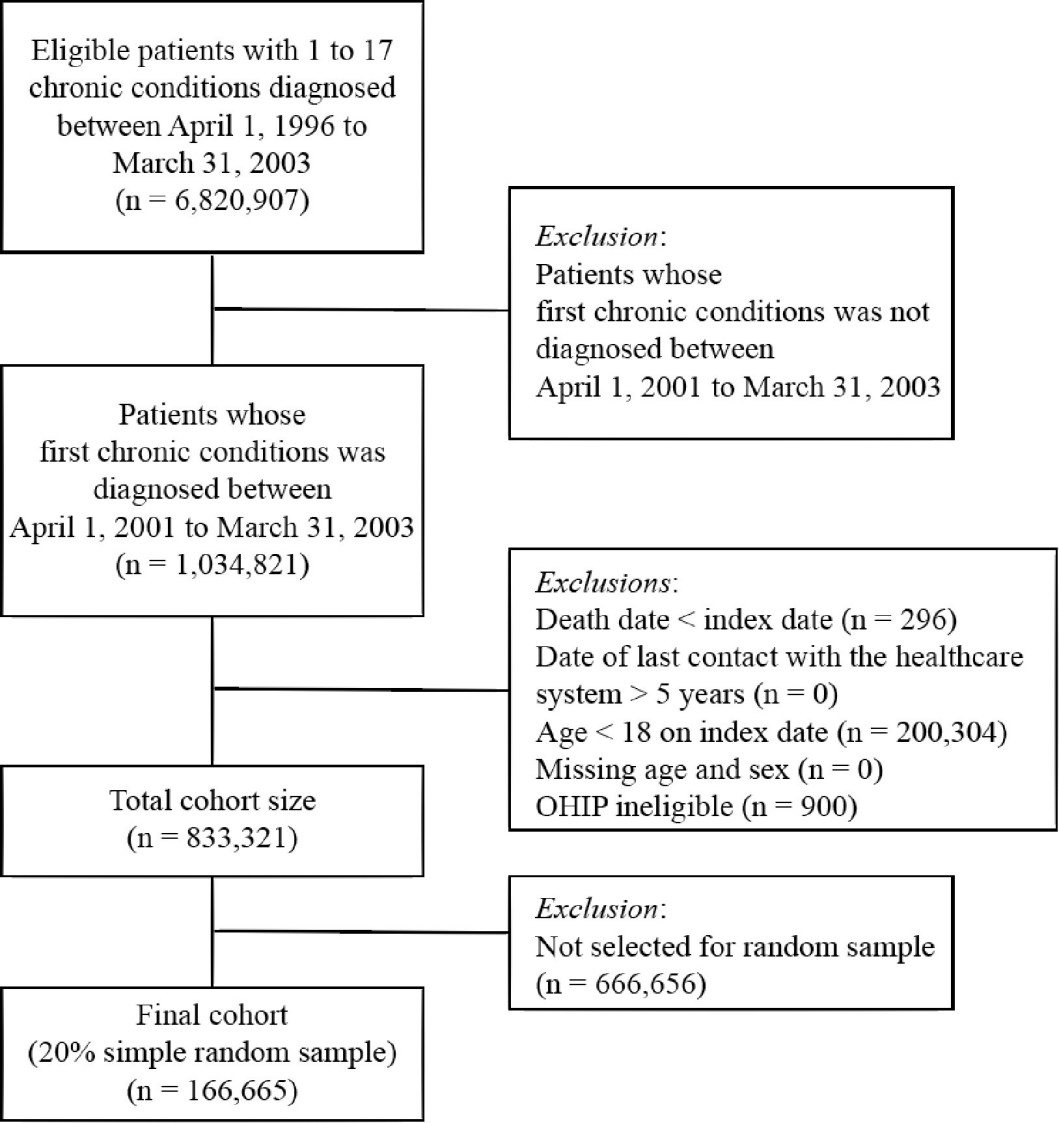
Selection of patients with a minimum of one out of seventeen chronic conditions for entry into the cohort (n = 166,665).

For all persons identified, we applied a look-back period that extended to April 1, 1996 and excluded individuals with any diagnosis made prior to April 1, 2001 to ensure only incident cases of multimorbidity were captured in the study follow-up period (n = 5,786,086). The earliest date that each patient was diagnosed with their first condition, in the period between 1 April 2001 and 31 March 2003, was identified as the index date. We excluded individuals with a registered date of death that preceded the date of their first diagnosis (n = 296), aged less than 18 years on the index date (n = 200,304), or were not eligible for the OHIP program (n = 900). The resultant population (n = 833,321) was reduced to a 20% random sample as each individual had up to 14 observations resulting in a very large database from which to attempt a longitudinal model with time-varying covariates in a constrained computing environment. Therefore, a still substantive 166,665 patients were included in our cohort. A patient flow diagram of the individuals that were part of the analyses with the inclusion criteria is demonstrated in Fig 1. S2 Table demonstrates the representativeness of the sample (S2 Table).

### Incident multimorbidity

Our primary outcome was the time in days from the index date to the diagnosis of any second chronic condition (amongst the 17 conditions under investigation) that was previously undiagnosed. The follow-up period extended from index date to March 31, 2015. We also quantified the time between the second and third, and third and fourth diagnoses for our secondary objectives.

### Continuity of care

We calculated continuity of care using the Bice-Boxerman Continuity of Care Index (COCI), a frequently used measure that is based on the number of visits made to each physician visited by a patient [42]. We chose this quantitative index because of its relevance to patients with multimorbidity who may receive care from many different types of physicians for the management of their chronic conditions [26]. This measure also accounts for physician referrals, wherein visits to physicians based on a referral are attributed to the referring physician.

COCI values range from 0 to 1, with zero indicating that all of a patient’s visits are to a different unreferred physician and one denotes perfect continuity or that all visits were with (or referred by) the same physician [13]. We categorized a patient as having high continuity if they had a continuity score that was greater than the median continuity score of the year prior to the diagnosis of the first condition and low continuity otherwise, which is consistent with previous studies [9,11,12]. A limitation to this measure is that it captures relationship continuity across visits and is only measured for individuals with at least 2 physician encounters (as relationship continuity is not a relevant construct for individuals with only one physician visit). We include individuals with no continuity of care measure in a mutually exculsive category of <2 visits.

### Confounders

The Registered Persons Database was used to collect information on individuals’ age at index in five year increments up to and including ≥ 80, sex (male/female), and place of residence (rural/urban). Specifically, patients are assigned the same rural or urban status as the census-defined geographical subdivision in which they reside by the Rurality Index of Ontario 2008 [43]. Neighourhood-income quintile was defined by linking postal codes to Statistics Canada income data where quintile 1 contains the lowest-income neighborhoods [44]. Although most physicians in Ontario are paid by fee-for-service payments, primary care enrolment models offering capitation were implemented in Ontario beginning in 2003. These models may provide an incentive for more wholistic care and be related to continuity. We collected information on patient enrolment in a primary care model from the Corporate Provider Database and Client Agency Program Enrolment databases [45]. The models included Family Health Groups (larger fee-for-service physicians with some capitation), Family Health Network or Organizations (primarily capitation), Family Health Teams (capitation with additional budgets for allied health professionals in a team-based model), ‘Other’ models of compensation (Comprehensive Care Model, Community Health Group, Community Sponsored Agreement, Group Health Centre, Health Services Organization, Primary Care Network, Rural and Northern Group, South Eastern Area Medical Organization, and St. Joseph’s Health Centre), and ‘not-enrolled’ (primarily small practice fee-for-service physicians). The annual number of inpatient and outpatient general practice and specialist visits were also identified from OHIP (measured in units of 5 visits/year for regression analyses) were also identified from OHIP because these are associated with both outcomes and exposure and capture the raw association between the number of physician visits with the likelihood of a new diagnosis.

### Statistical analyses

#### Main analyses

We summarized the sociodemographic characteristics and healthcare utilization of the cohort at index, and at the time of second and third diagnosis, using frequencies and proportions for categorical covariates, and means and standard deviations for continuous measures. The median follow-up time and interquartile ranges (IQR), and a simple count of the number diagnosed conditions during the follow-up period was also described.

We estimated the effect of COC on the rate at which an incremental chronic condition was diagnosed in the subsequent year using a multivariable cause-specific hazard regression model to account for death as a competing risk. This was necessary to avoid violations of informative and random censoring assumptions [46]. We treated COC as a time-dependent exposure following Andersen and Gill’s counting process method to avoid the bias that results from holding the value of continuity fixed at baseline [47–49]. To mitigate the possibility of reverse causation whereby a new diagnosis may lead patients to visit physicians more often, we used the lagged measure of COC based on physician visits in the prior year and then looked forward through the next year to observe any new diagnosis for all patients throughout the follow-up.

The association between continuity and the time to a second chronic condition was quantified with the cause-specific hazard ratio (CHR) adjusted for age, sex, neighborhood income, place of residence, primary care enrolment, and the number of visits to general practice and specialist physicians that provided care in inpatient and outpatient settings. With the exception of sex, all potential confounders were treated as time-dependent and measured in the year prior to the observation period. Patients were considered at-risk for multimorbidity from the date of diagnosis of their first chronic condition until they were diagnosed with a second condition or they were right-censored if death occurred, they lost OHIP eligibility, or the end of the follow-up period on March 31, 2015 was reached, whichever came first.

We also estimated the effect of continuity on the rate of diagnosis of a third and fourth condition following the same approach to determine whether continuity remained consistent at higher levels of multimorbidity. Patients were only considered at-risk for subsequent conditions from the date of diagnosis of their second and third condition where applicable, respectively. To assess the robustness of our primary findings to usual ambulatory care we also calculated COC using only outpatient visits and after excluding physicians primarily involved in interpretation of diagnostic tests, excluding any visits to specialist anaesthesiologists, diagnostic radiologists, and pathology and laboratory medicine physicians. This is consistent with prior studies of continuity using the Bice Index that include only in-person Assessment and Evaluation visits whereas these latter providers generally do not have ongoing individual consultations with a patient given the underlying nature of their discipline.[8]

#### Sensitivity analyses

We performed additional sensitivity analyses to determine the robustness of our results. First, we further enhanced the stability of the COCI index by calculating continuity only for patients with three or more visits to any physician annually [9,50]. We then testsed an alternative measure of continuity by replacing the COCI with the Usual Provider of Care (UPC) index, which simply measures the proportion of visits that a patient makes to their usual provider [51]. In this study, a patient’s usual provider was considered the physician that the patient visited most often each year. UPC scores ranged from 0 indicating no continuity to 1 or perfect continuity. We categorized patients with UPC scores above 0.75 as having high continuity as the distribution of UPC scores was strongly right-skewed [52,53]. Similar to the COCI analyses we included an indicator for patients who had fewer than 2 physician visits. Finally we excluded physician visits in the 30 days prior to a new diagnosis to mitigate the natural association of physician encounters and incident diagnoses. All tests of hypotheses were two-sided and significance was evaluated at the α = 0.05 level. We completed all statistical analyses using SAS version 9.4 (SAS Institute, Cary, North Carolina).

Ethics approval for this study was granted by the Health Sciences Research Ethics Board at the University of Toronto (#34619). Informed consent from patients was not necessary since health administrative data is de-identified prior to the conduct of research following provincial law.

## Results

### Baseline characteristics of patients

We identified n = 166,665 patients with a chronic condition in the set that were eligible for inclusion in our cohort. Table 1 provides summary population characteristics stratified by levels of continuity. Prior to the diagnosis of their first condition, 37% (n = 64,051) and 38% (n = 61,552) of patients had high and low continuity scores, 25% had fewer than 2 visits. Younger age groups were generally over-represented in the low-continuity group with older adults more common in the high-continuity group. Just over half of the cohort was female, and 88% of patients resided in an urban area at the time of their first diagnosis. There was a relatively equal representation by neighborhood income quintile, with each quintile containing approximately one fifth of the patients. Because the implementation of primary care enrolment models in Ontario began in 2004, the majority of individuals (98%) were not initially enrolled with a physician in a primary care model in 2003. We observed that general practice physicians in outpatient care were seen an average of five times annually, whereas fewer visits were made to specialists prior to index.

**Table 1:** Baseline sociodemographic characteristics and healthcare utilization of patients with two chronic conditions stratified by continuity of care (n = 166,665).

Characteristic	Low continuity‡ (n = 64,051)	High continuity (n = 61,552)	Missing continuity (n = 41,062)	All patients (n = 166,665)
**Age (years), n (%)**				
18 to 24	9431 (14.72)	4017 (6.53)	4014 (9.78)	17,462 (10.48)
25 to 29	6767 (10.57)	3939 (6.40)	3515 (8.56)	14,221 (8.53)
30 to 34	7417 (11.58)	5133 (8.34)	4263 (10.38)	16,813 (10.09)
35 to 39	8238 (12.86)	6393 (10.39)	5019 (12.22)	19,650 (11.79)
40 to 44	7385 (11.53)	7174 (11.66)	5392 (13.13)	19,951 (11.97)
45 to 49	6117 (9.55)	6773 (11.00)	4935 (12.02)	17,825 (10.70)
50 to 54	5023 (7.84)	6739 (10.95)	4060 (9.89)	15,822 (9.49)
55 to 59	3829 (5.98)	5418 (8.80)	2995 (7.29)	12,242 (7.35)
60 to 64	2869 (4.48)	4480 (7.28)	2207 (5.37)	9556 (5.73)
65 to 69	2327 (3.63)	3861 (6.27)	1705 (4.15)	7893 (4.74)
70 to 74	1844 (2.88)	3283 (5.33)	1260 (3.07)	6387 (3.83)
75 to 79	1291 (2.02)	2195 (3.57)	811 (1.98)	4297 (2.58)
≥ 80	1513 (2.36)	2147 (3.49)	886 (2.16)	4546 (2.73)
**Sex, n (%)**				
Male	28,757 (44.90)	26,962 (43.80)	24,873 (60.57)	80,592 (48.36)
Female	35,294 (55.10)	34,590 (56.20)	16,189 (39.43)	86,073 (51.64)
**Residence, n (%)**				
Rural	7917 (12.36)	7442 (12.09)	5235 (12.75)	20,594 (12.36)
Urban	56,134 (87.64)	54,110 (87.91)	35,827 (87.25)	146,071 (87.64)
**Neighborhood income quintile, n (%)**				
Quintile 1 (lowest income)	12,793 (19.97)	11,727 (19.05)	8460 (20.60)	32,980 (19.79)
Quintile 2	13,013 (20.32)	12,611 (20.49)	8336 (20.30)	33,960 (20.38)
Quintile 3	12,621 (19.70)	12,530 (20.36)	7817 (19.04)	32,968 (19.78)
Quintile 4	13,078 (20.42)	12,658 (20.56)	8400 (20.46)	34,136 (20.48)
Quintile 5 (highest income)	12,546 (19.59)	12,026 (19.54)	8049 (19.60)	32,621 (19.57)
**Primary care enrolment model, n (%)**				
Not-enrolled	62,567 (97.68)	59,947 (97.39)	39,989 (97.39)	162,503 (97.50)
Family Health Group	0 (0.00)	0 (0.00)	0 (0.00)	0 (0.00)
Family Health Team	0 (0.00)	0 (0.00)	0 (0.00)	0 (0.00)
Family Health Organization	0 (0.00)	0 (0.00)	0 (0.00)	0 (0.00)
Other†	246 (0.38)	368 (0.60)	217 (0.53)	4162 (2.50)
**Annual inpatient general practice visits, mean (SD)**	0.16 (1.48)	0.10 (1.59)	0.00087 (0.029)	0.10 (1.33)
**Annual outpatient general practice visits, mean (SD)**	5.76 (4.75)	6.57 (5.54)	0.44 (0.49)	4.75 (5.12)
**Annual inpatient specialist visits, mean (SD)**	0.10 (1.17)	0.01 (0.40)	0.00073 (0.027)	0.04 (0.77)
**Annual outpatient specialist visits, mean (SD)**	0.95 (1.85)	0.29 (1.18)	0.034 (0.18)	0.48 (1.41)
**No. of deaths, n(%)**	1445 (2.25)	1276 (2.07)	581 (1.41)	3302 (1.98)
**No. who received a positive chronic condition diagnosis, n(%)**	45,788 (71.48)	46,573 (75.66)	27,159 (66.14)	119,520 (71.71)

Abbreviations: IQR = Interquartile range; SD = Standard deviation.

Note: Chronic conditions considered were osteo- and other arthritis, osteoporosis, renal failure, cardiac arrhythmia, coronary artery disease, non-psychotic mood and anxiety disorders, other mental health conditions (including schizophrenia, delusions, and other psychoses, personality disorders, and substance abuse), dementia, rheumatoid arthritis, chronic obstructive pulmonary disease, congestive heart failure, acute myocardial infarction, asthma, hypertension, diabetes, stroke, or any cancer. The columns from left to right correspond to the characteristics of patients prior to the diagnosis of the first, second, and third chronic conditions, respectively. Age, sex, rural/urban residence, neighborhood income, and primary care enrolment model were determined at the beginning of the year prior to the development of these conditions.

‡Continuity of care was calculated with the Bice-Boxerman Index using all inpatient and outpatient (office, home, long-term care, emergency department, telephone, ‘undefined’) general practice physicians and specialist visits that were made throughout the year prior to the development of each consecutive condition. High versus low continuity was delineated by the median value of continuity among all patients at index.

†Family Health Networks, Comprehensive Care Model, Community Sponsored Agreement, Community Health Group, Group Health Center, Health Services Organization, Primary Care Network, Rural and Northern Group, South Eastern Area Medical Organization, and St. Joseph’s Health Centre.

### Median follow-up time and simple count of chronic conditions

The average total follow-up period was 12.8 years or a total of more than 2 million person-years of data. The median follow-up time was 1738 (IQR = 560, 4353), 1374 (IQR = 489, 2766), and 1019 (IQR = 362, 2071) days for the time to diagnosis of the second, third, and fourth chronic condition. The frequency of each subsequent diagnosis is reported in S3, S4 and S5 Tables with total population in each table (119 520, 68 021, and 34 089) representing the number of individuals diagnosed with a second, third, and fourth condition respectively. Mood disorders, arthritis, and cancer were the most commonly diagnosed as a first and second condition (S3 Table). Although most diagnoses of a third condition were still either arthritis or cancer, the incidence of hypertension was greater than mood disorders (S4 Table). This was also observed for the fourth condition except that the incidence of cancer was greater than that of arthritis (S5 Table).

In the baseline cohort with one condition (Table 1), death occurred prior to a second diagnosis more often amongst those with low continuity (n = 1445, 2.25%) as compared to those with high continuity (n = 1276, 2.07%) or those with fewer than 2 visits (n = 581, 1.41%). Similar patterns were observed and are reported in supplementary tables (S6 and S7 Tables) which contain descriptions of the cohort prior to the diagnosis of their third and fourth conditions respectively. Follow-up was terminated due to death for a total of n = 10,400 individuals in the cohort across the entire follow-up, with 3302 (1.98%), 3650 (3.05%), and 3448 (5.06%) individuals dying prior the diagnosis of a second, third, and fourth condition respectively. For regressions predicting the second, third and fourth condition, a total of 6078 (3.64%), 2091 (1.74%), and 681 (1.00%) of patients were right-censored due to the loss of eligibility for health insurance prior to developing a subsequent condition (data not shown).

### Multivariable cause-specific hazards regression of relationship continuity and morbidity

Among patients with one chronic condition, individuals with high continuity had an 8% lower rate of being diagnosed with a second chronic condition or multimorbidity relative to those with low continuity (adjusted CHR = 0.92; 95% CI = 0.91 to 0.93; *p*<0.0001) (Table 2). The subsequent rate of diagnosis of a third condition in patients with two conditions was also 8% lower in those with high continuity (0.92; 95% CI = 0.90 to 0.94; *p*<0.0001). We observed that patients with three conditions had an 9% lower rate of being diagnosed with a fourth condition when they had high continuity (0.91; 95% CI = 0.89 to 0.93; *p*<0.0001). The indicator for patient-period observations with fewer than 2 visits shows that those individuals who rarely went to their physician were unlikely to be diagnosed with a new condition in the subsequent period.

**Table 2:** Adjusted cause-specific hazard ratios of time-dependent continuity of care calculated with all inpatient and outpatient family physician and specialist visits (COC using ≥ 2 visits annually).

	Condition 1 (n = 166,665) to Condition 2			Condition 2 (n = 119,520) to Condition 3			Condition 3 (n = 68,021) to Condition 4		
**Characteristic**	CHR	95% CI	*p-value*	CHR	95% CI	*p-value*	CHR	95% CI	*p-value*
**Continuity of care‡**									
**Low (≤ 0.50)**	Reference	-	-	Reference	-	-	Reference	-	-
**High (> 0.50)**	0.92	(0.91 to 0.93)	<0.0001	0.92	(0.90 to 0.94)	<0.0001	0.91	(0.89 to 0.93)	<0.0001
**<2 visits**	0.73	(0.72 to 0.75)	<0.0001	0.62	(0.60 to 0.64)	<0.0001	0.59	(0.55 to 0.64)	<0.0001
**Age (years)**									
**18 to 24**	Reference	-	-	Reference	-	-	Reference	-	-
**25 to 29**	1.09	(1.04 to 1.13)	<0.0001	1.21	(1.09 to 1.35)	<0.0001	1.49	(1.07 to 2.07)	<0.0001
**30 to 34**	1.23	(1.19 to 1.28)	<0.0001	1.33	(1.20 to 1.48)	<0.0001	1.53	(1.11 to 2.10)	<0.0001
**35 to 39**	1.44	(1.39 to 1.49)	<0.0001	1.61	(1.45 to 1.78)	<0.0001	1.98	(1.45 to 2.71)	<0.0001
**40 to 44**	1.61	(1.56 to 1.67)	<0.0001	1.78	(1.62 to 2.00)	<0.0001	2.46	(1.81 to 3.36)	<0.0001
**45 to 49**	1.90	(1.84 to 1.98)	<0.0001	2.17	(1.97 to 2.40)	<0.0001	2.78	(2.04 to 3.79)	<0.0001
**50 to 54**	2.18	(2.10 to 2.26)	<0.0001	2.43	(2.21 to 2.68)	<0.0001	3.18	(2.33 to 4.33)	<0.0001
**55 to 59**	2.39	(2.30 to 2.48)	<0.0001	2.66	(2.41 to 2.93)	<0.0001	3.47	(2.55 to 4.73)	<0.0001
**60 to 64**	2.68	(2.58 to 2.79)	<0.0001	2.97	(2.69 to 3.27)	<0.0001	3.84	(2.82 to 5.24)	<0.0001
**65 to 69**	2.96	(2.85 to 3.08)	<0.0001	3.19	(2.89 to 3.52)	<0.0001	4.20	(3.08 to 5.72)	<0.0001
**70 to 74**	3.14	(3.01 to 3.27)	<0.0001	3.41	(3.09 to 3.77)	<0.0001	4.65	(3.41 to 6.33)	<0.0001
**75 to 79**	3.46	(3.31 to 3.61)	<0.0001	3.78	(3.42 to 4.19)	<0.0001	5.17	(3.80 to 7.05)	<0.0001
**≥ 80**	3.72	(3.56 to 3.88)	<0.0001	4.34	(3.92 to 4.79)	<0.0001	6.13	(4.50 to 8.35)	<0.0001
**Sex**									
**Male**	Reference	-	-	Reference	-	-	Reference	-	-
**Female**	1.01	(0.99 to 1.08)	0.36	0.97	(0.95 to 0.98)	<0.0001	0.90	(0.88 to 0.93)	<0.0001
**Residence**									
**Rural**	Reference	-	-	Reference	-	-	Reference	-	-
**Urban**	1.07	(1.05 to 1.09)	<0.0001	1.05	(1.03 to 1.08)	0.0001	1.01	(0.97 to 1.05)	0.63
**Neighborhood income quintile**									
**Quintile 1 (lowest income)**	Reference	-	-	Reference	-	-	Reference	-	-
**Quintile 2**	0.96	(0.94 to 0.98)	<0.0001	0.95	(0.92 to 0.98)	<0.0001	0.95	(0.92 to 0.99)	0.025
**Quintile 3**	0.93	(0.92 to 0.95)	<0.0001	0.94	(0.91 to 0.96)	<0.0001	0.94	(0.90 to 0.98)	0.0013
**Quintile 4**	0.92	(0.90 to 0.94)	<0.0001	0.93	(0.90 to 0.95)	<0.0001	0.94	(0.90 to 0.98)	0.0019
**Quintile 5 (highest income)**	0.90	(0.89 to 0.92)	<0.0001	0.90	(0.87 to 0.92)	<0.0001	0.91	(0.87 to 0.95)	<0.0001
**Primary care enrolment model**									
**Not-enrolled**	Reference	-	-	Reference	-	-	Reference	-	-
**Family Health Group**	1.12	(1.10 to 1.13)	<0.0001	1.01	(0.98 to 1.03)	0.61	1.03	(0.99 to 1.06)	0.13
**Family Health Network/Organization**	1.02	(1.00 to 1.04)	0.12	0.92	(0.90 to 0.95)	<0.0001	0.95	(0.91 to 0.99)	0.023
**Family Health Team**	0.99	(0.96 to 1.02)	0.38	0.90	(0.88 to 0.93)	<0.0001	0.91	(0.86 to 0.95)	<0.0001
** Other†**	1.11	(1.06 to 1.16)	<0.0001	1.02	(0.97 to 1.07)	0.37	1.04	(0.97 to 1.11)	0.30
**Inpatient general practice visits**	1.01	(1.00 to 1.01)	<0.0001	1.02	(1.00 to 1.03)	0.027	1.02	(1.01 to 1.04)	0.0029
**Inpatient specialist visits**	1.00	(0.99 to 1.01)	0.98	1.05	(1.03 to 1.07)	<0.0001	1.02	(0.99 to 1.05)	0.19
**Outpatient general practice visits**	1.02	(1.02 to 1.02)	<0.0001	1.10	(1.10 to 1.11)	<0.0001	1.08	(1.07 to 1.09)	<0.0001
**Outpatient specialist visits**	1.02	(1.02 to 1.02)	<0.0001	1.11	(1.09 to 1.12)	<0.0001	1.08	(1.07 to 1.09)	<0.0001

Note: The relationship between continuity and a) Time in days until the diagnosis of the 2nd condition among those with at least 1 condition (n = 166,665), b) Time in days until the diagnosis of the 3rd condition among those with at least 2 conditions (n = 119,520), and c) Time in days until the diagnosis of the 4th condition among those with at least 3 conditions (n = 68,021) was estimated with multivariable cause-specific hazards regression models. The effect estimate of continuity was adjusted for age, sex, neighborhood income, primary care enrolment model, number of physician visits (inpatient general practice, inpatient specialist, outpatient general practice, outpatient specialist), and place of residence simultaneously.

‡Continuity of care was measured using the Bice-Boxerman Index and categorized as high versus low continuity at the median among all patients at index. All visits to family physicians and specialists in outpatient settings (office, home, long-term care, emergency department, telephone, ‘undefined’) in the previous year were counted in the calculation of continuity.

†Comprehensive Care Model, Community Sponsored Agreement, Community Health Group, Group Health Center, Health Services Organization, Primary Care Network, Rural and Northern Group, South Eastern Area Medical Organization, and St. Joseph’s Health Centre.

Patient sociodemographic risk factors followed known patterns of risk for multimorbidity. A positive dose-response relationship was observed between age and multimorbidity. Patients aged 25–29 years had a 9% higher rate of multimorbidity (1.09; 1.04 to 1.13; *p*<0.0001), whereas the rate of diagnosis was almost four times that of 18–24 year olds for those aged 80 and above (3.72; 3.56 to 3.88; *p*<0.0001). The magnitude of the association of age with the rate of diagnosis was markedly stronger in patients with additional morbidity. Sex was not signficiantly associated with multimorbidity but females had a lower rate of third (0.97; 0.95 to 0.98; *p* < 0.0001), and fourth (0.90; 0.88 to 0.93; p<0.0001) diagnosis as compared with males. Income was inversely associated with the rate of a multimorbidity diagnosis such that patients in the highest income group had a 10% lower diagnosis rate (0.90; 0.89 to 0.92; *p*<0.0001) relative to patients with the lowest level of income consistently across all incremental diagnoses. Residing in urban areas was associated with a 7% higher rate of multimorbidity (1.07; 1.05 to 1.09; *p*<0.0001) with decreasing magnitude at higher levels of multimorbidity.

Being enrolled to a physician compensated through a blended capitation model in a Family Health Organization or Network was not associated with multimorbidity but was associated with an 8% reduction in the risk of a third condition (0.92; 0.90 to 0.95; *p =* 0.0001) and a 5% reduction in risk for a fourth condition (0.95; 0.91 to 0.99; p = 0.023) compared to patients cared for by primary care physicians who practiced in fee-for-service models. Patients enrolled in Family Health Teams with capitation and access to additional allied health professionals similarly experienced a 10% reduction in risk of a third (0.90; 0.88 to 0.93; *p* = 0.0001) and a 9% reduction in risk of a fourth condition (0.91; 0.86 to 0.95; p = 0.0001).

The cause-specific hazard ratio estimates for physician visits measures correspond to increments of five visits. There was a 2% increase in the rate of a diagnosis with multimorbidity for every five GP or specialist visits that occurred in an outpatient setting. This rose to between 8% and 11% increase in risk of a third and fourth condition.

We observed that the results from our main analyses were robust to changes in the definition of continuity when changing the physicians included in the COCI calculations. High continuity of care (greater than the median of 0.71) with outpatient-based general practitioners and specialists remained protective against the diagnosis of additional morbidity as shown in Table 3. Similarly, high continuity (greater than median of 0.50) was protective when specialists with limited patient interactions were excluded in Table 4.

**Table 3:** Adjusted cause-specific hazard ratios of time-dependent continuity of care calculated with all outpatient family physician and specialist visits (COC using ≥ 2 visits annually).

	Condition 1 (n = 166,665) to Condition 2			Condition 2 (n = 119,520) to Condition 3				Condition 3 (n = 68,021) to Condition 4		
**Characteristic**	CHR	95% CI	*p-value*	CHR	95% CI	*p-value*		CHR	95% CI	*p-value*
**Continuity of care‡**										
Low (≤ 0.71)	Reference	-	-	Reference	-	-		Reference	-	-
High (> 0.71)	0.91	(0.90 to 0.93)	<0.0001	0.91	(0.89 to 0.93)	<0.0001		0.93	(0.91 to 0.96)	<0.0001
<2 visits	0.75	(0.74 to 0.76)	<0.0001	0.67	(0.65 to 0.69)	<0.0001		0.68	(0.64 to 0.71)	<0.0001
**Age (years)**										
18 to 24	Reference	-	-	Reference	-	-		Reference	-	-
25 to 29	1.09	(1.05 to 1.13)	<0.0001	1.22	(1.10 to 1.36)	<0.0001		1.49	(1.07 to 2.07)	0.017
30 to 34	1.24	(1.20 to 1.30)	<0.0001	1.34	(1.21 to 1.50)	<0.0001		1.54	(1.12 to 2.11)	<0.0001
35 to 39	1.44	(1.39 to 1.50)	<0.0001	1.61	(1.46 to 1.78)	<0.0001		1.98	(1.45 to 2.71)	<0.0001
40 to 44	1.62	(1.57 to 1.68)	<0.0001	1.79	(1.62 to 1.97)	<0.0001		2.46	(1.80 to 3.36)	<0.0001
45 to 49	1.92	(1.86 to 1.99)	<0.0001	2.18	(1.98 to 2.41)	<0.0001		2.78	(2.04 to 3.79)	<0.0001
50 to 54	2.21	(2.13 to 2.29)	<0.0001	2.46	(2.23 to 2.71)	<0.0001		3.18	(2.34 to 4.34)	<0.0001
55 to 59	2.42	(2.34 to 2.51)	<0.0001	2.69	(2.44 to 2.97)	<0.0001		3.48	(2.56 to 4.74)	<0.0001
60 to 64	2.73	(2.63 to 2.83)	<0.0001	3.02	(2.74 to 3.33)	<0.0001		3.86	(2.84 to 5.26)	<0.0001
65 to 69	3.03	(2.91 to 3.14)	<0.0001	3.26	(2.95 to 3.60)	<0.0001		4.25	(3.12 to 5.79)	<0.0001
70 to 74	3.22	(3.10 to 3.35)	<0.0001	3.51	(3.17 to 3.87)	<0.0001		4.73	(3.47 to 6.44)	<0.0001
75 to 79	3.54	(3.40 to 3.70)	<0.0001	3.90	(3.52 to 4.31)	<0.0001		5.27	(3.86 to 7.18)	<0.0001
≥ 80	3.80	(3.65 to 4.00)	<0.0001	4.43	(4.01 to 4.90)	<0.0001		6.27	(4.61 to 8.54)	<0.0001
**Sex**										
Male	Reference	-	-	Reference	-	-		Reference	-	-
Female	1.01	(1.00 to 1.02)	0.058	0.97	(0.95 to 0.99)	0.0006		0.90	(0.88 to 0.93)	<0.0001
**Residence**										
Rural	Reference	-	-	Reference	-	-		Reference	-	-
Urban	1.04	(1.02 to 1.06)	<0.0001	1.02	(1.00 to 1.05)	0.11		0.99	(0.95 to 1.03)	0.47
**Neighborhood income quintile**										
Quintile 1 (lowest income)	Reference	-	-	Reference	-	-		Reference	-	-
Quintile 2	0.96	(0.95 to 0.98)	<0.0001	0.95	(0.92 to 0.98)	0.0002		0.95	(0.92 to 0.99)	0.019
Quintile 3	0.94	(0.92 to 0.95)	<0.0001	0.94	(0.91 to 0.96)	<0.0001		0.94	(0.90 to 0.98)	0.0016
Quintile 4	0.92	(0.90 to 0.94)	<0.0001	0.93	(0.90 to 0.95)	<0.0001		0.94	(0.90 to 0.98)	0.0021
Quintile 5 (highest income)	0.91	(0.89 to 0.92)	<0.0001	0.90	(0.87 to 0.92)	<0.0001		0.91	(0.87 to 0.95)	<0.0001
**Primary care enrolment model**										
Not-Enrolled	Reference	-	-	Reference	-	-		Reference	-	-
Family Health Group	1.11	(1.09 to 1.14)	0.63	1.01	(0.98 to 1.03)	0.54		1.03	(0.99 to 1.06)	0.15
Family Health Network or Organization	1.04	(1.02 to 1.07)	<0.0001	0.94	(0.91 to 0.97)	<0.0001		0.96	(0.92 to 1.01)	0.09
Family Health Team	1.02	(0.99 to 1.05)	<0.0001	0.93	(0.90 to 0.96)	<0.0001		0.93	(0.89 to 0.97)	0.0019
Other†	1.13	(1.08 to 1.18)	0.27	1.03	(0.98 to 1.08)	0.24		1.04	(0.97 to 1.11)	0.26
**Outpatient general practice visits**	1.13	(1.11 to 1.12)	<0.0001	1.11	(1.11 to 1.12)	<0.0001		1.09	(1.08 to 1.09)	<0.0001
**Outpatient specialist visits**	1.13	(1.10 to 1.17)	<0.0001	1.09	(1.06 to 1.12)	<0.0001	1.12		(1.08 to 1.16)	<0.0001

Abbreviations: CI = Confidence Interval; CHR = Cause-Specific Hazard Ratio.

Note: The relationship between continuity and a) Time in days until the diagnosis of the 2^nd^ condition among those with at least 1 condition (n = 166,665), b) Time in days until the diagnosis of the 3^rd^ condition among those with at least 2 conditions (n = 119,520), and c) Time in days until the diagnosis of the 4^th^ condition among those with at least 3 conditions (n = 68,021) was estimated with multivariable cause-specific hazards regression models. The effect estimate of continuity was adjusted for age, sex, neighborhood income, primary care enrolment model, number of physician visits (outpatient general practice, outpatient specialist), and place of residence simultaneously.

‡Continuity of care was measured using the Bice-Boxerman Index and categorized as high versus low continuity at the median among all patients at index. All visits to family physicians and specialists in outpatient settings (office, home, long-term care, emergency department, telephone, ‘undefined’) in the previous year were counted in the calculation of continuity.

†Comprehensive Care Model, Community Sponsored Agreement, Community Health Group, Group Health Center, Health Services Organization, Primary Care Network, Rural and Northern Group, South Eastern Area Medical Organization, and St. Joseph’s Health Centre.

**Table 4:** Adjusted cause-specific hazard ratios of time-dependent continuity of care calculated with all inpatient and outpatient family physician and specialist visits excluding visits to anaesthesiologists, diagnostic radiologists, and pathologists (COC using ≥ 2 visits annually).

	Condition 1 (n = 166,665) to Condition 2			Condition 2 (n = 119,520) to Condition 3			Condition 3 (n = 68,021) to Condition 4		
Characteristic	CHR	95% CI	*p-value*	CHR	95% CI	*p-value*	CHR	95% CI	*p-value*
**Continuity of care‡**									
Low (≤ 0.50)	Reference	-	-	Reference	-	-	Reference	-	-
High (> 0.50)	0.93	(0.92 to 0.94)	<0.0001	0.91	(0.90 to 0.93)	<0.0001	0.91	(0.89 to 0.94)	<0.0001
<2 visits	0.73	(0.72 to 0.74)	<0.0001	0.62	(0.60 to 0.64)	<0.0001	0.60	(0.56 to 0.64)	<0.0001
**Age (years)**									
18 to 24	Reference	-	-	Reference	-	-	Reference	-	-
25 to 29	1.09	(1.05 to 1.13)	<0.0001	1.22	(1.09 to 1.36)	<0.0001	1.49	(1.07 to 2.07)	<0.0001
30 to 34	1.24	(1.20 to 1.29)	<0.0001	1.34	(1.21 to 1.49)	<0.0001	1.53	(1.11 to 2.10)	<0.0001
35 to 39	1.45	(1.40 to 1.50)	<0.0001	1.62	(1.46 to 1.79)	<0.0001	1.99	(1.46 to 2.73)	<0.0001
40 to 44	1.62	(1.57 to 1.68)	<0.0001	1.80	(1.63 to 1.98)	<0.0001	2.48	(1.82 to 3.39)	<0.0001
45 to 49	1.92	(1.85 to 2.00)	<0.0001	2.19	(1.99 to 2.42)	<0.0001	2.80	(2.06 to 3.82)	<0.0001
50 to 54	2.20	(2.12 to 2.28)	<0.0001	2.46	(2.23 to 2.71)	<0.0001	3.20	(2.35 to 4.76)	<0.0001
55 to 59	2.40	(2.32 to 2.49)	<0.0001	2.69	(2.44 to 2.96)	<0.0001	3.49	(2.57 to 4.76)	<0.0001
60 to 64	2.70	(2.60 to 2.80)	<0.0001	3.00	(2.72 to 3.31)	<0.0001	3.87	(2.84 to 5.27)	<0.0001
65 to 69	2.98	(2.87 to 3.09)	<0.0001	3.23	(2.93 to 3.56)	<0.0001	4.23	(3.10 to 5.76)	<0.0001
70 to 74	3.16	(3.04 to 3.29)	<0.0001	3.45	(3.12 to 3.81)	<0.0001	4.68	(3.43 to 6.37)	<0.0001
75 to 79	3.47	(3.32 to 3.62)	<0.0001	3.82	(3.46 to 4.23)	<0.0001	5.19	(3.81 to 7.08)	<0.0001
≥ 80	3.72	(3.56 to 3.88)	<0.0001	4.35	(3.94 to 4.81)	<0.0001	6.14	(4.51 to 8.36)	<0.0001
**Sex**									
Male	Reference	-	-	Reference	-	-	Reference	-	-
Female	1.01	(1.00 to 1.03)	<0.0001	0.98	(0.96 to 0.99)	0.004	0.91	(0.89 to 0.93)	<0.0001
**Residence**									
Rural	Reference	-	-	Reference	-	-	Reference	-	-
Urban	1.07	(1.05 to 1.09)	<0.0001	1.05	(1.03 to 1.08)	0.0001	1.01	(0.97 to 1.05)	0.65
**Neighborhood income quintile**									
Quintile 1 (lowest income)	Reference	-	-	Reference	-	-	Reference	-	-
Quintile 2	0.96	(0.95 to 0.98)	<0.0001	0.95	(0.92 to 0.98)	0.0003	0.96	(0.92 to 0.99)	0.028
Quintile 3	0.94	(0.92 to 0.95)	<0.0001	0.94	(0.91 to 0.96)	<0.0001	0.94	(0.90 to 0.98)	0.0017
Quintile 4	0.92	(0.90 to 0.94)	<0.0001	0.93	(0.90 to 0.95)	<0.0001	0.94	(0.90 to 0.98)	0.0028
Quintile 5 (highest income)	0.91	(0.89 to 0.92)	<0.0001	0.90	(0.88 to 0.93)	<0.0001	1.04	(0.97 to 1.11)	0.30
**Primary care enrolment model**									
Not-enrolled	Reference	-	-	Reference	-	-	Reference	-	-
Family Health Group	1.11	(1.01 to 1.13)	<0.0001	1.01	(0.98 to 1.03)	0.61	1.03	(0.99 to 1.06)	0.13
Family Health Network or Organization	1.02	(1.00 to 1.04)	0.12	0.92	(0.90 to 0.95)	<0.0001	0.95	(0.91 to 0.99)	0.02
Family Health Team	0.99	(0.96 to 1.02)	0.46	0.91	(0.88 to 0.94)	<0.0001	0.91	(0.87 to 0.95)	<0.0001
Other†	1.11	(1.07 to 1.16)	<0.0001	1.03	(0.98 to 1.08)	0.29	1.04	(0.97 to 1.11)	0.30
**Inpatient general practice visits**	1.05	(1.03 to 1.06)	<0.0001	1.02	(1.01 to 1.04)	0.003	1.03	(1.01 to 1.04)	0.0008
**Inpatient specialist visits**	1.01	(0.99 to 1.04)	0.30	1.07	(1.04 to 1.09)	<0.0001	1.03	(1.00 to 1.05)	0.05
**Outpatient general practice visits**	1.11	(1.10 to 1.11)	<0.0001	1.10	(1.09 to 1.10)	<0.0001	1.08	(1.07 to 1.08)	<0.0001
**Outpatient specialist visits**	1.15	(1.13 to 1.17)	<0.0001	1.12	(1.10 to 1.14)	<0.0001	1.13	(1.11 to 1.15)	<0.0001

Abbreviations: CI = Confidence Interval; CHR = Cause-Specific Hazard Ratio.

Note: The relationship between continuity and a) Time in days until the diagnosis of the 2^nd^ condition among those with at least 1 condition (n = 166,665), b) Time in days until the diagnosis of the 3^rd^ condition among those with at least 2 conditions (n = 119 520), and c) Time in days until the diagnosis of the 4^th^ condition among those with at least 3 conditions (n = 68 021) was estimated with multivariable cause-specific hazards regression models. The effect estimate of continuity was adjusted for age, sex, neighborhood income, primary care enrolment model, number of physician visits (inpatient general practice, inpatient specialist, outpatient general practice, outpatient specialist), and place of residence simultaneously.

‡Continuity of care was measured using the Bice-Boxerman Index and categorized as high versus low continuity at the median among all patients at index. All visits to family physicians and specialists in outpatient settings (office, home, long-term care, emergency department, telephone, ‘undefined’) in the previous year were counted in the calculation of continuity, excluding visits to anaesthesiologists, diagnostic radiologists, and pathologists.

†Comprehensive Care Model, Community Sponsored Agreement, Community Health Group, Group Health Center, Health Services Organization, Primary Care Network, Rural and Northern Group, South Eastern Area Medical Organization, and St. Joseph’s Health Centre.

#### Sensitivity analyses

There were no changes to the effect estimates when continuity was only calculated for patients with three or more physician visits annually per year (S8 Table). When the COCI was replaced with the UPC, the rate of diagnosis of multimorbidity and subsequent conditions was still lower for individuals with high relative to low continuity by 10–13% (S9 Table). Our final sensitivity analysis excluded from the COC calculation visits in the 30 days prior to a new diagnosis to assess the potential impact of visits to new specialists or other physicians leading to new diagnoses. Only 3% of all physician visits occurred within the 30 days prior to disease diagnosis and this analysis had no impact on observed effects from the baseline model.

## Discussion

### Principal findings

We observed that high relationship continuity of care with physicians was associated with a lower rate of diagnosis with a second chronic condition or multimorbidity in the year following continuity measurement. High relationship continuity was further associated with a lower risk of diagnosis of the next incremental chronic condition with an increasing number of conditions. The findings were robust to changes in the definition of continuity and sample populations. These results indicate that receiving more care from a primary physician or physicians referred by that primary physician in one year is associated with a lower risk of a new diagnosis in the subsequent year. Given that just over a quarter of all individuals progressed to two conditions and more than forty percent of the latter group then progressed to a third condition, an 8% reduction in the risk of acquiring a new diagnosis over our 14-year observation period is substantial. If policy and practice can shift more individuals toward higher continuity of care, we can expect a concomitant decrease in the number of new diagnoses and decreases in the associated health care burden to patients and costs to the system.

### Comparisons with other studies

To our knowledge, the only studies that have previously examined the association between continuity of care and multimorbidity were from the United Kingdom [24,25]. Gulliford et al. (2012) examined the association between multiple long-term chronic conditions and the odds of experiencing problems with relationship continuity. They found that those with zero long-term conditions were most likely to have difficulties with establishing continuity [25]. Salisbury et al. (2011) noted that continuity with physicians and nurses is inversely related to multimorbidity although the inclusion of two different types of providers in their calculation of a single continuity score creates uncertainty regarding which provider was responsible for the observed effect [24]. Importantly, both cross-sectional analyses cannot be readily compared with our study because they each treated continuity as an outcome rather than specifying it as an exposure. This is the first study to explore the association between continuity and the occurrence of morbidity longitudinally.

Relationship continuity is influenced by several factors and is founded upon the decisions and willingness of both patients and physicians to seek or recommend additional care. In Ontario, primary care physicians provide a gatekeeper role and referrals are necessary for specialists to receive full payment from government. An array of factors determine the strength and sustainability of continuity which are not necessarily specific to Ontario including physician reimbursement mechanisms, physician accessibility, and an individual patient’s own proclivity towards building a trusting relationship or seeking additional opinions.

While death as an outcome was not the primary interest in our analyses, we observed that the proportion of individuals who died increased with the number of diagnoses. This finding is consistent with prior work that has linked increased mortality risk to additional development of conditions, the onset of which can trigger worsening severity of conditions and declines in physiological reserve [3,54]. It also justifies our use of a competing risk framework for our analyses.

### Potential mechanisms underlying the protective effect of relationship continuity

There are several potential explanations for how continuity is associated with fewer future diagnoses of chronic conditions among patients with pre-existing illness. One possibility is that patients who are not exposed to new physicians have missed diagnoses while patients who seek out new (unreferred) physicians are found to have diagnoses that were missed by their usual care providers. Another possibility is that high continuity of care encourages the delivery of more preventive care services which lowers the rate of diagnoses of multimorbidity. For example, Medicaid enrollees in the United States with their own personal physician and a usual source of care were screened for abnormal blood pressure and cholesterol and had a routine physical examination more often than those who did not [55]. Another explanation is that high continuity encourages the adoption of healthier lifestyle behaviours which then delays the decline in health. When patients have confidence in a usual source of care for their health problems, preventive care, and referrals to other professionals, they were found to be more likely to obtain advice on smoking, diet, and exercise relative to those who did not [56,57]. Finally, it is hypothesized that the effect of continuity on the rate of multimorbidity diagnoses may be mediated by limiting polypharmacy or the concomitant use of multiple medications and potential associated adverse drug events [11,12]. There is robust evidence which demonstrates that many chronic conditions are induced by medications. For example, anti-cancer, hematologic, neurological and psychiatric, pulmonary, and rheumatological medications are each able to induce heart failure [58,59]. Consistent with this mechanism, multimorbid patients are known to be subject to extensive treatment burden from medications. Such burden may be related to the absence of appropriate clinical practice guidelines that address multimorbidity, the involvement of multiple providers that care for a patient, and the lack of regular medication reviews by a physician [16,26,60]. Obtaining a better understanding of the specific mediators including polypharmacy, through which continuity acts to lower the rate of diagnoses should be the subject of future studies to facilitate quality improvement efforts in primary care.

### Implications for policymakers and physicians

With increasing multimorbidity unfolding in Canada and around the world, there is a growing need for interventions that are aimed at reducing the incidence of chronic conditions. The positive impact of relationship continuity of care with physicians on the health of patients is increasingly a focus of research [61,62]. The present longitudinal study provides suggestive evidence that continuity could also be considered protective against new diagnoses of chronic conditions across all ages. Indeed, while multimorbidity disproportionately affects the elderly, previous studies have shown that the absolute number of patients with multimorbidity is higher amongst the non-elderly which underscores the importance of adopting a life-course approach to the prevention of multimorbidity [61,63,64]. Efforts to improve continuity in various care settings should involve general practitioners as the clinical lead because of their patient-centred approach to care. In particular, the emphasis that general practitioners place on a patient’s preferences, values, self-management abilities, and other non-disease related contextual factors is essential for the management of chronic conditions in the absence of known cures [65–68]. It may be argued that caution should be exercised regarding the extent to which continuity is implemented in practice.

A recognized disadvantage of relationship continuity is clinical inertia or the failure of a practitioner to escalate treatment to achieve the recommended goals for their patient [69]. As a result, when high continuity or always visiting the same pracititioner prevents a diagnosis from being made patients may be denied the necessary treatments that would potentially alter the clinical course of their illness. This underscores the importance of achieving a balance between seeing the same practitioner consistently and other providers as required for care. Additional research studying the influence of referred and unreferred physician visits and resulting diagnoses and outcomes are required to study these dynamic relationships.

### Strengths and limitations of the study

A strength of this study was the large multimorbidity cohort that we established by leveraging Ontario’s population-based health administrative data. The twelve-year population based study provides the most extensive follow-up of multimorbid patients to date compared with the few cohort studies that have been performed on multimorbidity [70]. The COCI measure not only counts visits to an individual physician, as in the UPC, but also attributes referred visits to other physicians back to the referring physician. Ontario’s single-payer health insurance system uses a gatekeeper model and some visits may not be paid for if there was no recorded referral from another physician. Our modelling approach allowed the variables within each of the models to vary over time. This allowed for physician utilization and continuity of care measures to change with changes in patient health needs and healthcare utilization, particularly as patients become diagnosed with a new condition which may require additional follow-up visits and consultations with specialists. We therefore avoided the bias that is associated with using baseline-fixed covariates when time-dependent covariates are more appropriate [47,48]. Moreover, this investigation was enhanced with the time to event model and explicit consideration of death as a competing risk. Overall, the study design and population based data that we used likely supports the generalizability of our findings on continuity to patients with chronic conditions living elsewhere with healthcare systems that provide universal healthcare coverage.

There are several limitations worth highlighting. First, we cannot distinguish among the constructs of informational and management continuity that may be closely intertwined with relationship continuity. Second, we cannot rule out confounding by other variables that we were unable to measure in the administrative data. The simple count that we used to measure multimorbidity is one of the most common methods, but it does not address the confounding caused by the severity of an individual’s illness. Continuity of care may also have differential effects among individuals with particular combinations of disease. Ethnicity was also not accounted for although patients belonging to different ethnic groups experience different levels of continuity and rates of multimorbidity [71]. Third, we were only able to measure the time until the diagnosis of a condition rather than when an individual first developed a condition. Fourth, since we included a limited subset of all known chronic conditions, other diagnosed conditions that were not on the list may confound the association between continuity and the rate of multimorbidity diagnoses. Fifth, only eight of the conditions included in this study were ascertained with validated algorithms, whereas the remaining conditions were identified with consistent but non-validated algorithm. Sixth, the OHIP database only records encounters with physicians who submit fee for-service claims for service reimbursement to the provincial government thereby excluding any visits to salaried physicians [45]. However these physicians are few in number in Ontario and are identified within ‘other’ primary care models which was not significant in our results. Seventh, the possibility of spurious relationships and reverse causation cannot be entirely refuted using an observational study design, however well-constructed.

## Conclusion

Physician and patient continuity is an enduring value of primary based care with relevance in specialist care settings as well. Its importance will become even more pronounced with the passage of time as existing health systems across the world shift toward more patient-centric ideals. Part of this will be driven by rapid and currently ongoing changes in the clinical population which is trending towards more complex and likely older patients. At the same time we also anticipate that tomorrow’s patient will be more informed and demand enhanced provisions and quality from their caretakers.

In this observational study, we found that the benefit of relationship continuity goes beyond merely improving quality and the care experience, We found that it impacts the rate of diagnosis of multimorbidity and this association is sustained across multiple definitions of continuity including different types of physician visits and through increasing levels of multimorbidity. If delayed diagnosis protects against over-diagnosis and over-treatment or acts to improve patient engagement in health-promoting activity, our study suggests that relationship continuity should be pursued for the management of patients with multimorbidity when appropriate [61,63,64].

## Supporting information

S1 Table ICD diagnostic codes for chronic conditions. (DOCX)

S2 Table Comparison of random sample with full population. (DOCX)

S3 Table Counts of conditions as a second diagnosis among patients with one condition at baseline. (DOCX)

S4 Table Counts of conditions as a third diagnosis among patients with two conditions. (DOCX)

S5 Table Counts of conditions as a fourth diagnosis among patients with three conditions. (DOCX)

S6 Table Baseline sociodemographic characteristics and healthcare utilization of patients with two chronic conditions stratified by continuity of care. (DOCX)

S7 Table Baseline sociodemographic characteristics and healthcare utilization of patients with three chronic conditions stratified by continuity of care. (DOCX)

S8 Table Adjusted cause-specific hazard ratios with continuity defined with three or more visits. (DOCX)

S9 Table Adjusted cause-specific hazard ratios with continuity calculated using UPC. (DOCX)

S1 File STROBE statement—checklist of items that should be included in reports of observational studies. (DOCX)
