# Supplementary material for: Association between continuity of care and subsequent diagnosis of multimorbidity in Ontario, Canada from 2001–2015: A retrospective cohort study
Source: PLoS One. 2021 Mar 11;16(3):e0245193. doi: 10.1371/journal.pone.0245193 (PMC7951913; doi:10.1371/journal.pone.0245193)
Supplement: S1 Table — (DOCX) [file pone.0245193.s001.docx]

S1 Table. List of 17 chronic conditions and the corresponding ICD-8/9/10 diagnostic codes.

| **Chronic Condition** | **ICD-9** | **ICD-10-CA** | **ODB** |
| --- | --- | --- | --- |
| **Acute Myocardial Infarction** | 410 | I21, I22 |  |
| **Arthritis (Osteoarthritis, Synovitis, Fibrositis, Connective tissue disorders, Ankylosing spondylitis, Gout Traumatic arthritis, pyogenic arthritis, Joint derangement, Dupuytren’s contracture, Other MSK disorders)** | 710, 711, 715, 716, 718, 720, 727, 728, 729, 739, 274 | M15-M19, M00-M03, M07, M10, M11-M14, M20-M25, M30-M36, M65-M79 |  |
| **Asthma** | 493 | J45 |  |
| **Cancer (all)** | 140-239 | C00-C26, C30-C44, C45-C97 |  |
| **Cardiac arrhythmias** | DAD: 427.3/OHIP: 427 | I48.0, I48.1 |  |
| **Chronic coronary syndrome (excluding acute myocardial infarction)** | 411-414 | I20, I22-125 |  |
| **Chronic obstructive pulmonary disease** | 491, 492, 496 | J41, J43, J44 |  |
| **Congestive heart failure** | 428 | 150.0, 150.1, 150.9 |  |
| **Dementia** | DAD: 046.1, 290.0, 290.1, 290.2, 290.3, 290.4, 294, 331.0, 331.1, 331.5, F331.82/OHIP: 290, 331 | F00, F01, F02, F03, G30 | Cholinesterase Inhibitors |
| **Diabetes mellitus** | 250 | E08 – E13 |  |
| **Hypertension** | 401, 402, 403, 404, 405 | I10, I11, I12, I13, I15 |  |
| **Mood disorders (Anxiety, depression, and other non-psychotic disorders)** | 296, 300, 309, 311 | F30, F31, F32, F33, F34 (excl. F34.0), F38, F39, F40, F41, F42, F43.1, F43.2, F43.8, F44, F45.0, F45.1, F45.2, F48, F53.0, F68.0, F93.0, F99 |  |
| **(Other) Mental health conditions** | 291, 292, 295, 297, 298, 299, 301, 302, 303, 304, 305, 306, 307, 313, 314, 315, 319 | F04, F050, F058, F059, F060, F061, F062, F063, F064, F07, F08, F10, F11, F12, F13, F14, F15, F16, F17, F18, F19, F20, F21, F22, F23, F24, F25, F26, F27, F28, F29, F340, F35, F36, F37, F430, F439, F453, F454, F458, F46, F47, F49, F50, F51, F52, F531, F538, F539, F54, F55, F56, F57, F58, F59, F60, F61, F62, F63, F64, F65, F66, F67, F681, F688, F69, F70, F71, F72, F73, F74, F75, F76, F77, F78, F79, F80, F81, F82, F83, F84, F85, F86, F87, F88, F89, F90, F91, F92, F931, F932, F933, F938, F939, F94, F95, F96, F97, F98 |  |
| **Osteoporosis** | 733 | M81, M82 |  |
| **Renal failure** | 403, 404, 584, 585, 586, v451 | N17, N18, N19, T82.4, Z49.2, Z99.2 |  |
| **Rheumatoid Arthritis** | 714 | M05, M06 |  |
| **Stroke (excluding transient ischemic attack)** | 430, 431, 432, 434, 436 | I60-I64 |  |

Abbreviations: ICD = International Classification of Diseases, 9th and 10th Revision, Canadian Modification (ICD-10-CA), ODB = Ontario Drug Benefit Claims Database.
