# Supplementary material for: Association between continuity of care and subsequent diagnosis of multimorbidity in Ontario, Canada from 2001–2015: A retrospective cohort study
Source: PLoS One. 2021 Mar 11;16(3):e0245193. doi: 10.1371/journal.pone.0245193 (PMC7951913; doi:10.1371/journal.pone.0245193)
Supplement: S2 Table — (DOCX) [file pone.0245193.s002.docx]

S2 Table. Population characteristics compared to 20% random sample.

| **Characteristic** | **Full Population**  **(n = 833,321)** | **Random 20% Sample**  **(n = 166,665)** | **p-value** |
| --- | --- | --- | --- |
| **Continuity of care‡** |  |  |  |
| Low (≤ 0.50) | 319,184 (38.3%) | 64,051 (38.4%) | 0.339 |
| High (> 0.50) | 309,342 (37.1%) | 61,552 (36.9%) |  |
| < 2 MD visits | 204,795 (24.6%) | 41,062 (24.6%) |  |
| **Age (years)** |  |  |  |
| 18 to 24 | 87,093 (10.5%) | 17,462 (10.5%) | 0.913 |
| 25 to 29 | 71,248 (8.5%) | 14,221 (8.5%) |  |
| 30 to 34 | 84,201 (10.1%) | 16,813 (10.1%) |  |
| 35 to 39 | 97,405 (11.7%) | 19,650 (11.8%) |  |
| 40 to 44 | 99,349 (11.9%) | 19,951 (12.0%) |  |
| 45 to 49 | 89,637 (10.8%) | 17,825 (10.7%) |  |
| 50 to 54 | 79,605 (9.6%) | 15,822 (9.5%) |  |
| 55 to 59 | 61,541 (7.4%) | 12,242 (7.3%) |  |
| 60 to 64 | 47,980 (5.8%) | 9,556 (5.7%) |  |
| 65 to 69 | 39,669 (4.8%) | 7,893 (4.7%) |  |
| 70 to 74 | 31,364 (3.8%) | 6,387 (3.8%) |  |
| 75 to 79 | 21,756 (2.6%) | 4,297 (2.6%) |  |
| ≥ 80 | 22,473 (2.7%) | 4,546 (2.7%) |  |
| **Sex** |  |  |  |
| Male | 402,433 (48.3%) | 80,592 (48.4%) | 0.638 |
| Female | 430,888 (51.7%) | 86,073 (51.6%) |  |
| **Residence** |  |  |  |
| Rural | 103,134 (12.4%) | 20,594 (12.4%) | 0.823 |
| Urban | 730,187 (87.6%) | 146,071 (87.6%) |  |
| **Income quintile** |  |  |  |
| Quintile 1 (lowest) | 164,487 (19.7%) | 32,980 (19.8%) | 0.297 |
| Quintile 2 | 168,048 (20.2%) | 33,960 (20.4%) |  |
| Quintile 3 | 165,750 (19.9%) | 32,968 (19.8%) |  |
| Quintile 4 | 171,522 (20.6%) | 34,136 (20.5%) |  |
| Quintile 5 (highest) | 163,514 (19.6%) | 32,621 (19.6%) |  |
| **Primary care enrolment model** |  |  |  |
| Not-enrolled | 812,450 (97.5%) | 162,503 (97.5%) | 0.861 |
| Other† | 20,871 (2.5%) | 4,162 (2.5%) |  |
| **Inpatient PC visits** | 0.10 ± 1.22 | 0.10 ± 1.34 | 0.217 |
| **Inpatient Spec visits** | 0.05 ± 0.81 | 0.05 ± 0.77 | 0.663 |
| **Outpatient PC visits** | 4.75 ± 5.15 | 4.75 ± 5.13 | 0.743 |
| **Outpatient Spec visits** | 0.48 ± 1.44 | 0.48 ± 1.42 | 0.930 |

PC= Primary Care; Spec.=Specialist;

‡Continuity of care was measured using the Bice-Boxerman Index and categorized as high versus low continuity at the median among all patients at index. † Family Health Network, Community Sponsored Agreement, Community Health Group, Group Health Center, Health Services Organization, Primary Care Network, Rural and Northern Group, South Eastern Area Medical Organization, and St. Joseph’s Health Centre.
