# Supplementary material for: Association between continuity of care and subsequent diagnosis of multimorbidity in Ontario, Canada from 2001–2015: A retrospective cohort study
Source: PLoS One. 2021 Mar 11;16(3):e0245193. doi: 10.1371/journal.pone.0245193 (PMC7951913; doi:10.1371/journal.pone.0245193)
Supplement: S3 Table — (DOCX) [file pone.0245193.s003.docx]

S3 Table. Count and percentage of each condition occurring between 2001-2015 as a second diagnosis among patients with one condition at index (baseline).

|  | Condition 2 | | | | | | | | | | | | | | | | | |
| --- | --- | --- | --- | --- | --- | --- | --- | --- | --- | --- | --- | --- | --- | --- | --- | --- | --- | --- |
| Condition 1 | AMI | AR | Asthma | Cancer | CA | CCS | COPD | CHF | DEM | DM | HTN | MD | MHC | OP | RF | RA | Stroke | Total,  N (%) |
| AMI  (736/166,665) | NA | 10 (1.44) | - | 6  (0.86) | - | 575 (82.85) | - | 26 (3.75) | - | 10 (1.44) | 32 (4.61) | 10  (1.44) | - | 0  (0.00) | - | - | - | 694 (100.00) |
| AR  (37,751/166,665) | 270 (1.00) | NA | 950 (3.52) | 5473 (20.31) | 563 (2.09) | 864 (3.21) | 305 (1.13) | 215 (0.80) | 224 (0.83) | 2086 (7.74) | 5160 (19.15) | 7172 (26.61) | 2067 (7.67) | 793 (2.94) | 241  (0.89) | 359 (1.33) | 209 (0.78) | 26,951 (100.00) |
| Asthma  (5908/166,665) | - | 990 (23.13) | NA | 642 (15.00) | 67 (1.57) | 69 (1.61) | 125 (2.92) | 24 (0.56) | 18 (0.42) | 225 (5.26) | 536 (12.52) | 1227 (28.67) | 269  (6.29) | 50  (1.17) | 15  (0.35) | - | 16 (0.37) | 4280 (100.00) |
| Cancer  (23,110/166,665) | 119 (0.76) | 4292 (27.46) | 441 (2.82) | NA | 397 (2.54) | 424 (2.71) | 242 (1.55) | 211 (1.35) | 135 (0.86) | 828 (5.30) | 2631 (16.83) | 4203 (26.89) | 856  (5.48) | 473 (3.03) | 181  (1.16) | 37 (0.24) | 162 (1.04) | 15,632 (100.00) |
| CA  (2104/166,665) | - | 294 (16.70) | 28 (1.59) | 283 (16.07) | NA | 219 (12.44) | 23 (1.31) | 98 (5.57) | 37 (2.10) | 83 (4.71) | 247 (14.03) | 275 (15.62) | 73  (4.15) | 23  (1.31) | 25  (1.42) | - | 37 (2.10) | 1761 (100.00) |
| CCS  (4314/166,665) | 137 (3.59) | 652 (17.11) | 64 (1.68) | 440 (11.55) | 249 (6.53) | NA | 89 (2.34) | 160 (4.20) | 74 (1.94) | 297 (7.79) | 856 (22.46) | 417 (10.94) | 169  (4.43) | - | 85  (2.23) | - | 72 (1.89) | 3811 (100.00) |
| COPD  (1022/166,665) | - | 120 (13.47) | 93 (10.44) | 155 (17.40) | 38 (4.26) | 52 (5.84) | NA | 48 (5.39) | 14 (1.57) | 46 (5.16) | 124 (13.92) | 85  (9.54) | 42  (4.71) | 24  (2.69) | 22  (2.47) | - | 15 (1.68) | 891 (100.00) |
| CHF  (623/166,665) | 17 (3.20) | 56 (10.55) | 7  (1.32) | 39  (7.34) | 77 (14.50) | 115 (21.66) | 30 (5.65) | NA | 20 (3.77) | 24 (4.52) | 60 (11.30) | 30  (5.65) | 15  (2.82) | - | 20  (3.77) | - | 15 (2.82) | 531 (100.00) |
| DEM  (853/166,665) | 13 (2.30) | 61 (10.80) | 8  (1.42) | 64 (11.33) | 35 (6.19) | 33 (5.84) | 21 (3.72) | 33 (5.84) | NA | 29 (5.13) | 56 (9.91) | 112 (19.82) | 50  (8.85) | - | 11  (1.95) | - | 30 (5.31) | 565 (100.00) |
| DM  (7398/166,665) | 94 (1.62) | 1226 (21.12) | 114 (1.96) | 799 (13.77) | 134 (2.31) | 344 (5.93) | 60 (1.03) | 77 (1.33) | 55 (0.95) | NA | 1561 (26.90) | 812 (13.99) | 270  (4.65) | 80  (1.38) | 99  (1.71) | 12 (0.21) | 67 (1.15) | 5804 (100.00) |
| HTN  (21,042/166,665) | 207 (1.24) | 4147 (24.92) | 390 (2.34) | 2957 (17.77) | 463 (2.78) | 975 (5.86) | 201 (1.21) | 192 (1.15) | 201 (1.21) | 2365 (14.21) | NA | 2696 (16.20) | 858  (5.16) | 388 (2.33) | 293  (1.76) | 35 (0.21) | 274 (1.65) | 16,642 (100.00) |
| MD  (46,282/166,665) | 136 (0.45) | 8874 (29.63) | 1304 (4.35) | 6254 (20.88) | 458 (1.53) | 557 (1.86) | 249 (0.83) | 103 (0.34) | 220 (0.73) | 1475 (4.92) | 4057 (13.54) | NA | 5398 (18.02) | 487 (1.63) | 162  (0.54) | 67 (0.22) | 153 (0.51) | 29,954 (100.00) |
| MHC  (10,606/166,665) | 70 (0.87) | 1682 (20.90) | 213 (2.65) | 987 (12.27) | 91 (1.13) | 129 (1.60) | 122 (1.52) | 37 (0.46) | 72 (0.89) | 381 (4.74) | 876 (10.89) | 3205 (39.83) | NA | 76  (0.94) | 51  (0.63) | 9  (0.11) | 45 (0.56) | 8046 (100.00) |
| OP  (2762/166,665) | 7 (0.30) | 660 (28.70) | 44 (1.91) | 464 (20.17) | 53 (2.30) | 63 (2.74) | 35 (1.52) | 18 (0.78) | 49 (2.13) | 71 (3.09) | 416 (18.09) | 314 (13.65) | 68  (2.96) | NA | 14  (0.61) | 6  (0.26) | 18 (0.78) | 2300 (100.00) |
| RF  (717/166,665) | 8 (1.42) | 94 (16.70) | - | 101 (17.94) | 25 (4.44) | 47 (8.35) | 17 (3.02) | 35 (6.22) | 24 (4.26) | 29 (5.15) | 77 (13.68) | 56  (9.95) | 30  (5.33) | 7  (1.24) | NA | - | 8  (1.42) | 563 (100.00) |
| RA  (273/166,665) | 8 (3.24) | 112 (45.34) | - | 22 (8.91) | - | 6  (2.43) | - | - | - | 10 (4.05) | 29 (11.74) | 30  (12.15) | - | 6  (2.43) | - | NA | - | 247 (100.00) |
| Stroke  (1164/166,665) | 11 (1.30) | 115 (13.56) | 8  (0.94) | 124 (14.62) | 50 (5.90) | 50 (5.90) | 19 (2.24) | 39 (4.60) | 47 (5.54) | 48 (5.66) | 149 (17.57) | 126 (14.86) | 39  (4.60) | - | 16  (1.89) | - | NA | 848 (100.00) |
| Total, N(%) | 1124 (0.94) | 23,385 (19.57) | 3672 (3.07) | 18,810 (15.74) | 2716 (2.27) | 4522 (3.78) | 1543 (1.29) | 1318 (1.10) | 1193 (1.00) | 8007 (6.70) | 16,867 (14.11) | 20,770 (17.38) | 10,213 (8.55) | 2471 (2.07) | 1238 (1.04) | 543 (0.45) | 1128 (0.94) | 119,520 (100.00) |

Abbreviations: AMI = Acute myocardial infarction; AR = Arthritis; CA = Cardiac arrhythmia; CCS = Chronic coronary syndrome; COPD = Chronic obstructive pulmonary disease; CHF = Congestive heart failure; DEM = Dementia; DM =

Diabetes mellitus; HTN = Hypertension; MD = Mood disorders; MHC = Mental health conditions; NA = Not applicable; OP = Osteoporosis; RF = Renal failure; RA = Rheumatoid arthritis.

Note: % represent the row total (e.g. 1.44% of patients with acute myocardial infarction as their first chronic condition developed arthritis as their second condition). The first, second, and third most common 2^nd^ condition is highlighted in each

row with blue, green, and orange, respectively. The proportions in the first column represent the number of patients with 1 out of 17 conditions as their first condition (e.g. 736/166,665 patients were diagnosed with acute myocardial infarction as

their first condition). Empty cells indicated with ‘-‘ were censored due to privacy requirements.
