# Supplementary material for: Association between continuity of care and subsequent diagnosis of multimorbidity in Ontario, Canada from 2001–2015: A retrospective cohort study
Source: PLoS One. 2021 Mar 11;16(3):e0245193. doi: 10.1371/journal.pone.0245193 (PMC7951913; doi:10.1371/journal.pone.0245193)
Supplement: S4 Table — (DOCX) [file pone.0245193.s004.docx]

S4 Table. Count and percentage of each condition occurring as a third diagnosis between 2001 – 2015 among patients diagnosed with two conditions during the study period.

|  | Condition 3 | | | | | | | | | | | | | | | | | |
| --- | --- | --- | --- | --- | --- | --- | --- | --- | --- | --- | --- | --- | --- | --- | --- | --- | --- | --- |
| Condition 2 | AMI | AR | Asthma | Cancer | CA | CCS | COPD | CHF | DEM | DM | HTN | MD | MHC | OP | RF | RA | Stroke | Total,  N (%) |
| AMI  (1124/119,520) | NA | 18 (3.90) | - | 18 (3.90) | 10 (2.17) | 295 (63.99) | - | 35 (7.59) | - | 14 (3.04) | 30 (6.51) | 15  (3.25) | 8  (1.74) | - | - | - | - | 461 (100.00) |
| AR  (23,385/119,520) | 164 (1.26) | NA | 463  (3.56) | 2742 (21.07) | 421 (3.24) | 560 (4.30) | 217 (1.67) | 189 (1.45) | 177 (1.36) | 1288 (9.90) | 2287 (17.57) | 2023 (15.55) | 1408 (10.82) | 440 (3.38) | 246  (1.89) | 222 (1.71) | 166 (1.28) | 13 013 (100.00) |
| Asthma  (3672/119,520) | 14 (0.61) | 497 (21.75) | NA | 345 (15.10) | 33 (1.44) | 70 (3.06) | 110 (4.81) | 30 (1.31) | 15 (0.66) | 140 (6.13) | 364 (15.93) | 362 (15.84) | 225  (9.85) | 34  (1.49) | 21  (0.92) | 13 (0.57) | 12 (0.53) | 2285 (100.00) |
| Cancer  (18,810/119,520) | 107 (1.17) | 2115 (23.15) | 299  (3.27) | NA | 361 (3.95) | 400 (4.38) | 210 (2.30) | 165 (1.81) | 141 (1.54) | 713 (7.80) | 1579 (17.28) | 1564 (17.12) | 787  (8.61) | 277 (3.03) | 221  (2.42) | 46 (0.50) | 153 (1.67) | 9138 (100.00) |
| CA  (2716/119,520) | - | 243 (14.27) | 27  (1.59) | 272 (15.97) | NA | 182 (10.69) | 39 (2.29) | 138 (8.10) | 51 (2.99) | 100 (5.87) | 235 (13.80) | 193 (11.33) | 96  (5.64) | 30  (1.76) | 33  (1.94) | - | 42 (2.47) | 1703 (100.00) |
| CCS  (4522/119,520) | 109 (3.18) | 515 (15.02) | 63  (1.84) | 482 (14.06) | 247 (7.21) | NA | 97 (2.83) | 195 (5.69) | 70 (2.04) | 295 (8.61) | 672 (19.60) | 336 (9.80) | 149  (4.35) | 31  (0.90) | 80  (2.33) | 15 (0.44) | 72 (2.10) | 3428 (100.00) |
| COPD  (1543/119,520) | 17 (1.67) | 146 (14.30) | 67  (6.56) | 177 (17.34) | 43 (4.21) | 50 (4.90) | NA | 58 (5.68) | 29 (2.84) | 79 (7.74) | 132 (12.93) | 99  (9.70) | 51  (5.00) | 25  (2.45) | 28  (2.74) | 8  (0.78) | 12 (1.18) | 1021 (100.00) |
| CHF  (1318/119,520) | 29 (3.58) | 68 (8.40) | 16  (1.98) | 72 (8.89) | 101 (12.47) | 157 (19.38) | 50 (6.17) | NA | 30 (3.70) | 31 (3.83) | 83 (10.25) | 53  (6.54) | 33  (4.07) | - | 59  (7.28) | - | 20 (2.47) | 810 (100.00) |
| DEM (1193/119,520) | 18 (2.64) | 74 (10.83) | - | 87 (12.74) | 32 (4.69) | 42 (6.15) | 19 (2.78) | 40 (5.86) | NA | 29 (4.25) | 65 (9.52) | 115 (16.84) | 59  (8.64) | 16  (2.34) | 39  (5.71) | - | 44 (6.44) | 683 (100.00) |
| DM  (8007/119,520) | 85 (1.84) | 865 (18.71) | 114  (2.47) | 806 (17.43) | 132 (2.85) | 295 (6.38) | 69 (1.49) | 113 (2.44) | 79 (1.71) | NA | 889 (19.23) | 566 (12.24) | 323  (6.99) | 63  (1.36) | 131  (2.83) | 21 (0.45) | 73 (1.58) | 4624 (100.00) |
| HTN  (16,867/119,520) | 177 (1.73) | 1993 (19.50) | 274  (2.68) | 1832 (17.93) | 380 (3.72) | 677 (6.63) | 215 (2.10) | 194 (1.90) | 162 (1.59) | 1290 (12.62) | NA | 1418 (13.88) | 785  (7.68) | 253 (2.48) | 281  (2.75) | 60 (0.59) | 227 (2.22) | 10,218 (100.00) |
| MD  (20,770/119,520) | 113 (0.97) | 2548 (21.98) | 433  (3.74) | 2186 (18.86) | 292 (2.52) | 382 (3.30) | 196 (1.69) | 117 (1.01) | 238 (2.05) | 917 (7.91) | 1879 (16.21) | NA | 1690 (14.58) | 266 (2.29) | 164  (1.41) | 61 (0.53) | 111 (0.96) | 11,593 (100.00) |
| MHC  (10,213/119,520) | 50 (0.90) | 1397 (25.22) | 208  (3.75) | 918 (16.57) | 133 (2.40) | 130 (2.35) | 118 (2.13) | 41 (0.74) | 89 (1.61) | 418 (7.55) | 837 (15.11) | 975 (17.60) | NA | 70  (1.26) | 88  (1.59) | 12 (0.22) | 56 (1.01) | 5540 (100.00) |
| OP  (2471/119,520) | 10 (0.62) | 329 (20.32) | 33  (2.04) | 343 (21.19) | 51 (3.15) | 57 (3.52) | 24 (1.48) | - | 39 (2.41) | 80 (4.94) | 300 (18.53) | 214 (13.22) | 70  (4.32) | NA | 20  (1.24) | - | 15 (0.93) | 1619 (100.00) |
| RF  (1238/119,520) | 21 (2.70) | 131 (16.84) | 15  (1.93) | 130 (16.71) | 32 (4.11) | 61 (7.84) | 19 (2.44) | 53 (6.81) | 29 (3.73) | 49 (6.30) | 96 (12.34) | 66  (8.48) | 36  (4.63) | - | NA | - | 27 (3.47) | 778 (100.00) |
| RA  (543/119,520) | - | 53 (14.80) | 12  (3.35) | 73 (20.39) | 12 (3.35) | 10 (2.79) | - | - | - | 26 (7.26) | 68 (18.99) | 56 (15.64) | 17  (4.75) | 7  (1.96) | - | NA | - | 358 (100.00) |
| Stroke  (1128/119,520) | 12 (1.60) | 93 (12.42) | - | 86 (11.48) | 49 (6.54) | 60 (8.01) | 18 (2.40) | 27 (3.60) | 57 (7.61) | 42 (5.61) | 122 (16.29) | 99 (13.22) | 36  (4.81) | 14  (1.87) | 23  (3.07) | - | NA | 749 (100.00) |
| Total, N(%) | 947 (1.39) | 11,085 (16.30) | 2038 (3.00) | 10,569 (15.54) | 2329 (3.42) | 3428 (5.04) | 1410 (2.07) | 1426 (2.10) | 1215 (1.79) | 5511 (8.10) | 9638 (14.17) | 8154 (11.99) | 5773 (8.49) | 1542 (2.27) | 1441 (2.12) | 477 (0.70) | 1038 (1.53) | 68,021 (100.00) |

Abbreviations: AMI = Acute myocardial infarction; AR = Arthritis; CA = Cardiac arrhythmia; CCS = Chronic coronary syndrome; COPD = Chronic obstructive pulmonary disease; CHF = Congestive heart failure; DEM = Dementia; DM =

Diabetes mellitus; HTN = Hypertension; MD = Mood disorders; MHC = Mental health conditions; NA = Not applicable; OP = Osteoporosis; RF = Renal failure; RA = Rheumatoid arthritis.

Note: % represent the row total (e.g. 3.90% of patients with acute myocardial infarction as their first condition developed arthritis as their second condition). The first, second, and third most common 2^nd^ condition is highlighted in each row with

blue, green, and orange, respectively. The proportions in the first column represent the number of patients with 1 out of 17 conditions as their second condition (e.g. 1124/199,520 patients were diagnosed with acute myocardial infarction as their second condition). Empty cells indicated with ‘-‘ were censored due to privacy requirements.
