# Supplementary material for: Association between continuity of care and subsequent diagnosis of multimorbidity in Ontario, Canada from 2001–2015: A retrospective cohort study
Source: PLoS One. 2021 Mar 11;16(3):e0245193. doi: 10.1371/journal.pone.0245193 (PMC7951913; doi:10.1371/journal.pone.0245193)
Supplement: S5 Table — (DOCX) [file pone.0245193.s005.docx]

S5 Table. Count and percentage of each condition occurring as a fourth diagnosis between 2001 – 2015 among patients diagnosed with a third condition during the study period.

|  | Condition 4 | | | | | | | | | | | | | | | | | |
| --- | --- | --- | --- | --- | --- | --- | --- | --- | --- | --- | --- | --- | --- | --- | --- | --- | --- | --- |
| Condition 3 | AMI | AR | Asthma | Cancer | CA | CCS | COPD | CHF | DEM | DM | HTN | MD | MHC | OP | RF | RA | Stroke | Total,  N (%) |
| AMI  (947/68,021) | NA | 16 (4.08) | - | 17 (4.34) | 17 (4.34) | 222 (56.63) | 8  (2.04) | 35 (8.93) | - | 13 (3.32) | 17 (4.34) | 14 (3.57) | 10  (2.55) | - | 10  (2.55) | - | - | 392 (100.00) |
| AR  (11,085/68,021) | 91 (1.82) | NA | 167  (3.33) | 911 (18.19) | 242 (4.83) | 285 (5.69) | 136 (2.72) | 127 (2.54) | 120 (2.40) | 559 (11.16) | 788 (15.73) | 528 (10.54) | 488  (9.74) | 185 (3.69) | 174  (3.47) | 107 (2.14) | 101 (2.02) | 5009 (100.00) |
| Asthma  (2038/68,021) | 10 (0.87) | 221 (19.34) | NA | 193 (16.89) | 31 (2.71) | 38 (3.32) | 71 (6.21) | 32 (2.80) | 10 (0.87) | 93 (8.14) | 149 (13.04) | 130 (11.37) | 107  (9.36) | 15  (1.31) | 24  (2.10) | 6  (0.52) | 13 (1.14) | 1143 (100.00) |
| Cancer  (10,569/68,021) | 76 (1.74) | 721 (16.54) | 142  (3.26) | NA | 242 (5.55) | 216 (4.96) | 156 (3.58) | 175 (4.01) | 121 (2.78) | 456 (10.46) | 675 (15.49) | 493 (11.31) | 411  (9.43) | 127 (2.91) | 205  (4.70) | 31 (0.71) | 112 (2.57) | 4359 (100.00) |
| CA  (2329/68,021) | 16 (1.22) | 151 (11.54) | 32  (2.44) | 189 (14.44) | NA | 152 (11.61) | 34 (2.60) | 158 (12.07) | 58 (4.43) | 88 (6.72) | 125 (9.55) | 101 (7.72) | 86  (6.57) | 24  (1.83) | 54  (4.13) | 7  (0.53) | 34 (2.60) | 1309 (100.00) |
| CCS  (3428/68,021) | 82 (3.20) | 310 (12.11) | 44  (1.72) | 326 (12.74) | 204 (7.97) | NA | 82 (3.20) | 196 (7.66) | 61 (2.38) | 247 (9.65) | 437 (17.08) | 223 (8.71) | 133  (5.20) | 37  (1.45) | 110  (4.30) | 7  (0.27) | 60 (2.34) | 2559 (100.00) |
| COPD  (1410/68,021) | 24 (2.81) | 94 (10.99) | 68  (7.95) | 117 (13.68) | 35 (4.09) | 61 (7.13) | NA | 79 (9.24) | 34 (3.98) | 64 (7.49) | 109 (12.75) | 56 (6.55) | 46  (5.38) | - | 27  (3.16) | - | 23 (2.69) | 855 (100.00) |
| CHF  (1426/68,021) | 32 (3.44) | 88 (9.46) | 12  (1.29) | 82 (8.82) | 117 (12.58) | 159 (17.10) | 35 (3.76) | NA | 36 (3.87) | 64 (6.88) | 98 (10.54) | 51 (5.48) | 33  (3.55) | - | 79  (8.49) | - | 35 (3.76) | 930 (100.00) |
| DEM  (1215/68,021) | - | 53 (7.95) | - | 82 (12.29) | 41 (6.15) | 32 (4.80) | 30 (4.50) | 48 (7.20) | NA | 47 (7.05) | 59 (8.85) | 89 (13.34) | 58  (8.70) | 13  (1.95) | 48  (7.20) | - | 49 (7.35) | 667 (100.00) |
| DM  (5511/68,021) | 52 (2.02) | 407 (15.78) | 73  (2.83) | 473 (18.34) | 100 (3.88) | 176 (6.82) | 58 (2.25) | 81 (3.14) | 63 (2.44) | NA | 376 (14.58) | 268 (10.39) | 216  (8.38) | 44  (1.71) | 105  (4.07) | 26 (1.01) | 61 (2.37) | 2579 (100.00) |
| HTN  (9638/68,021) | 98 (2.00) | 737 (15.04) | 133  (2.71) | 819 (16.71) | 237 (4.84) | 368 (7.51) | 157 (3.20) | 145 (2.96) | 136 (2.78) | 642 (13.10) | NA | 540 (11.02) | 410  (8.37) | 120 (2.45) | 211  (4.31) | 40 (0.82) | 107 (2.18) | 4900 (100.00) |
| MD  (8154/68,021) | 56 (1.37) | 605 (14.76) | 142  (3.47) | 656 (16.01) | 161 (3.93) | 209 (5.10) | 110 (2.68) | 105 (2.56) | 179 (4.37) | 416 (10.15) | 594 (14.49) | NA | 508 (12.40) | 133 (3.25) | 102  (2.49) | 33 (0.81) | 89 (2.17) | 4098 (100.00) |
| MHC  (5773/68,021) | 31 (1.17) | 486 (18.40) | 110  (4.16) | 467 (17.68) | 90 (3.41) | 114 (4.31) | 70 (2.65) | 41 (1.55) | 63 (2.38) | 283 (10.71) | 401 (15.18) | 318 (12.04) | NA | 47  (1.78) | 68  (2.57) | 14 (0.53) | 39 (1.48) | 2642 (100.00) |
| OP  (1542/68,021) | 8  (0.95) | 133 (15.78) | 19  (2.25) | 127 (15.07) | 37 (4.39) | 33 (3.91) | 15 (1.78) | 18 (2.14) | 45 (5.34) | 65 (7.71) | 122 (14.47) | 114 (13.52) | 51  (6.05) | NA | 22  (2.61) | 14 (1.66) | 20 (2.37) | 843 (100.00) |
| RF  (1441/68,021) | 27 (3.24) | 113 (13.57) | 16  (1.92) | 114 (13.69) | 39 (4.68) | 58 (6.96) | 27 (3.24) | 102 (12.24) | 39 (4.68) | 62 (7.44) | 74 (8.88) | 76 (9.12) | 38  (4.56) | - | NA | - | 33 (3.96) | 833 (100.00) |
| RA  (477/68,021) | - | 22 (8.87) | 8  (3.23) | 61 (24.60) | 8 (3.23) | 10 (4.03) | 7  (2.82) | 9  (3.63) | - | 14 (5.65) | 42 (16.94) | 25 (10.08) | 12  (4.84) | 7  (2.82) | 11  (4.44) | NA | - | 248 (100.00) |
| Stroke  (1038/68,021) | 12 (1.66) | 66 (9.13) | - | 92 (12.72) | 34 (4.70) | 61 (8.44) | 31 (4.29) | 50 (6.92) | 80 (11.07) | 39 (5.39) | 78 (10.79) | 74 (10.24) | 46  (6.36) | 15  (2.07) | 37  (5.12) | - | NA | 723 (100.00) |
| Total, N(%) | 626 (1.84) | 4223 (12.39) | 981  (2.88) | 4726 (13.86) | 1635 (4.80) | 2194 (6.44) | 1027 (3.01) | 1401 (4.11) | 1057 (3.10) | 3152 (9.25) | 4144 (12.16) | 3100 (9.09) | 2653 (7.78) | 802 (2.35) | 1287 (3.78) | 296 (0.87) | 785 (2.30) | 34,089 (100.00) |

Abbreviations: AMI = Acute myocardial infarction; AR = Arthritis; CA = Cardiac arrhythmia; CCS = Chronic coronary syndrome; COPD = Chronic obstructive pulmonary disease; CHF = Congestive heart failure; DEM = Dementia; DM =

Diabetes mellitus; HTN = Hypertension; MD = Mood disorders; MHC = Mental health conditions; NA = Not applicable; OP = Osteoporosis; RF = Renal failure; RA = Rheumatoid arthritis.

Note: % represent the row total (e.g. 4.08% of patients with acute myocardial infarction as their first condition developed arthritis as their second condition). The first, second, and third most common 2^nd^ condition is highlighted in each row with

blue, green, and orange, respectively. The proportions in the first column represent the number of patients with 1 out of 17 conditions as their third condition (e.g. 947/68,021 patients were diagnosed with acute myocardial infarction as

their third condition). Empty cells indicated with ‘-‘ were censored due to privacy requirements
