# Supplementary material for: Association between continuity of care and subsequent diagnosis of multimorbidity in Ontario, Canada from 2001–2015: A retrospective cohort study
Source: PLoS One. 2021 Mar 11;16(3):e0245193. doi: 10.1371/journal.pone.0245193 (PMC7951913; doi:10.1371/journal.pone.0245193)
Supplement: S6 Table — (DOCX) [file pone.0245193.s006.docx]

**S6 Table.** Baseline sociodemographic characteristics and healthcare utilization of patients with two chronic conditions stratified by continuity of care (n = 119,520)

| Characteristic | Low  continuity‡  (n = 46,101) | High  continuity  (n = 52,529) | < 2 visits  (n = 20,890) | All patients  (n = 119,520) |
| --- | --- | --- | --- | --- |
| Age (years), n (%) |  |  |  |  |
| 18 to 24 | 2489 (5.40) | 1118 (2.13) | 630 (3.02) | 4237 (3.55) |
| 25 to 29 | 3222 (6.99) | 1878 (3.58) | 999 (4.78) | 6099 (5.10) |
| 30 to 34 | 4303 (9.33) | 3108 (5.92) | 1455 (6.97) | 8866 (7.42) |
| 35 to 39 | 5097 (11.06) | 4005 (7.62) | 1931 (9.24) | 11,033 (9.23) |
| 40 to 44 | 5451 (11.82) | 5448 (10.37) | 2588 (12.39) | 13,487 (11.28) |
| 45 to 49 | 5339 (11.58) | 6126 (11.66) | 2880 (13.79) | 14,345 (12.00) |
| 50 to 54 | 4756 (10.32) | 6412 (12.21) | 2807 (13.44) | 13,975 (11.69) |
| 55 to 59 | 4131 (8.96) | 5816 (11.07) | 2330 (11.15) | 12,277 (10.27) |
| 60 to 64 | 3214 (6.97) | 5112 (9.73) | 1721 (8.24) | 10,047 (8.41) |
| 65 to 69 | 2561 (5.56) | 4354 (8.29) | 1297 (6.21) | 8212 (6.87) |
| 70 to 74 | 2113 (4.58) | 3651 (6.95) | 890 (4.26) | 6654 (5.57) |
| 75 to 79 | 1596 (3.46) | 2659 (5.06) | 599 (2.87) | 4854 (4.06) |
| ≥ 80 | 1829 (3.97) | 2842 (5.41) | 763 (3.65) | 5434 (4.55) |
| Sex, n (%) |  |  |  |  |
| Male | 20,773 (45.06) | 23,667 (45.06) | 12,610 (60.36) | 57,050 (47.73) |
| Female | 25,328 (54.94) | 28,862 (54.94) | 8280 (39.64) | 62,470 (52.27) |
| Residence, n (%) |  |  |  |  |
| Rural | 5610 (12.17) | 6155 (11.72) | 2776 (13.29) | 14,541 (12.17) |
| Urban | 40,404 (87.64) | 46,275 (88.09) | 18,047 (86.39) | 104,726 (87.62) |
| Missing | 87 (0.19) | 99 (0.19) | 67 (0.32) | 253 (0.21) |
| Neighbourhood income quintile, n (%) |  |  |  |  |
| Quintile 1 (lowest income) | 8997 (19.52) | 9877 (18.80) | 4027 (19.28) | 22,901 (19.16) |
| Quintile 2 | 9103 (19.75) | 10,575 (20.13) | 4132 (19.78) | 23,810 (19.92) |
| Quintile 3 | 9104 (19.75) | 10,821 (20.60) | 4097 (19.61) | 24,022 (20.10) |
| Quintile 4 | 9390 (20.37) | 10,781 (20.52) | 4236 (20.28) | 24,407 (20.42) |
| Quintile 5 (highest income) | 9263 (20.09) | 10,260 (19.53) | 4266 (20.42) | 23,789 (19.90) |
| Missing | 244 (0.53) | 215 (0.41) | 132 (0.63) | 591 (0.49) |
| Primary care enrolment model, n (%) |  |  |  |  |
| Not-enrolled | 30,672 (66.53) | 32,862 (62.56) | 14,750 (70.61) | 78,284 (65.50) |
| Family Health Group | 8127 (17.63) | 10,932 (20.81) | 2495 (11.94) | 21,554 (18.03) |
| Family Health Team | 2776 (6.02) | 2982 (5.68) | 1455 (6.97) | 7213 (6.03) |
| Family Health Organization | 2515 (5.46) | 3012 (5.73) | 1401 (6.71) | 6928 (5.80) |
| Other† | 2011 (4.36) | 2741 (5.22) | 789 (3.78) | 5541 (4.64) |
| Annual inpatient general practice visits, mean (SD) | 0.28 (2.36) | 0.11 (1.68) | 0.00095 (0.030) | 0.15 (1.84) |
| Annual outpatient general practice visits, mean (SD) | 6.79 (5.70) | 7.62 (6.19) | 0.43 (0.49) | 6.04 (6.02) |
| Annual inpatient specialist visits, mean (SD) | 0.20 (1.84) | 0.023 (0.58) | 0.00071 (0.026) | 0.088 (1.21) |
| Annual outpatient specialist visits, mean (SD) | 1.43 (2.95) | 0.37 (1.42) | 0.038 (0.19) | 0.72 (2.14) |
| No. of deaths, n(%) | 1890 (4.09) | 1564 (2.97) | 196 (0.93) | 3650 (3.05) |
| No. who received a positive chronic condition diagnosis, n(%) | 30,119 (65.33) | 33,045 (62.90) | 4857 (23.25) | 68,021 (56.91) |

Abbreviations: IQR = Interquartile range; SD = Standard deviation.

Note: Chronic conditions considered were osteo- and other arthritis, osteoporosis, renal failure, cardiac arrhythmia, coronary artery disease, non-psychotic mood and anxiety disorders, other mental health conditions (including schizophrenia, delusions, and other psychoses, personality disorders, and substance abuse), dementia, rheumatoid arthritis, chronic obstructive pulmonary disease, congestive heart failure, acute myocardial infarction, asthma, hypertension, diabetes, stroke, or any cancer. The columns from left to right correspond to the characteristics of patients prior to the diagnosis of the first, second, and third chronic conditions, respectively. Age, sex, rural/urban residence, neighbourhood income, and primary care enrolment model were determined at the beginning of the year prior to the development of these conditions.

‡Continuity of care was calculated with the Bice-Boxerman Index using all inpatient and outpatient (office, home, long-term care, emergency department, telephone, ‘undefined’) general practice physicians and specialist visits that were made throughout the year prior to the development of each consecutive condition. High versus low continuity was delineated by the median value of continuity among all patients at index.

†Family Health Networks, Comprehensive Care Model, Community Sponsored Agreement, Community Health Group, Group Health Center, Health Services Organization, Primary Care Network, Rural and Northern Group, South Eastern Area Medical Organization, and St. Joseph’s Health Centre.
