# Supplementary material for: Association between continuity of care and subsequent diagnosis of multimorbidity in Ontario, Canada from 2001–2015: A retrospective cohort study
Source: PLoS One. 2021 Mar 11;16(3):e0245193. doi: 10.1371/journal.pone.0245193 (PMC7951913; doi:10.1371/journal.pone.0245193)
Supplement: S8 Table — (DOCX) [file pone.0245193.s008.docx]

S9 Table. Adjusted cause-specific hazard ratios of time-dependent continuity of care calculated with all inpatient and outpatient family physician and specialist visits (using ≥ 3 visits annually)

|  | **Condition 1 (n = 166,665) to Condition 2** | | | **Condition 2 (n = 119,520) to Condition 3** | | | **Condition 3 (n = 68,021) to**  **Condition 4** | | |
| --- | --- | --- | --- | --- | --- | --- | --- | --- | --- |
| **Characteristic** | CHR | 95% CI | *p-value* | CHR | 95% CI | *p-value* | CHR | 95% CI | *p-value* |
| **Continuity of care‡** |  |  |  |  |  |  |  |  |  |
| Low (≤ 0.50) | Reference | - | - | Reference | - | - | Reference | - | - |
| High (> 0.50) | 0.93 | (0.92 to 0.94) | <0.0001 | 0.92 | (0.91 to 0.94) | <0.0001 | 0.91 | (0.89 to 0.94) | <0.0001 |
| <3 visits | 0.75 | (0.74 to 0.76) | <0.0001 | 0.66 | (0.64 to 0.68) | <0.0001 | 0.66 | (0.63 to 0.70) | <0.0001 |
| **Age (years)** |  |  |  |  |  |  |  |  |  |
| 18 to 24 | Reference | - | - | Reference | - | - | Reference | - | - |
| 25 to 29 | 1.09 | (1.04 to 1.13) | <0.0001 | 1.21 | (1.09 to 1.35) | 0.0004 | 1.49 | (1.07 to 2.07) | 0.018 |
| 30 to 34 | 1.23 | (1.19 to 1.28) | <0.0001 | 1.33 | (1.20 to 1.47) | <0.0001 | 1.52 | (1.11 to 2.09) | 0.001 |
| 35 to 39 | 1.44 | (1.39 to 1.49) | <0.0001 | 1.60 | (1.45 to 1.77) | <0.0001 | 1.98 | (1.45 to 2.71) | <0.0001 |
| 40 to 44 | 1.61 | (1.56 to 1.67) | <0.0001 | 1.78 | (1.61 to 1.96) | <0.0001 | 2.46 | (1.80 to 3.36) | <0.0001 |
| 45 to 49 | 1.90 | (1.84 to 1.97) | <0.0001 | 2.16 | (1.96 to 2.38) | <0.0001 | 2.78 | (2.04 to 3.79) | <0.0001 |
| 50 to 54 | 2.18 | (2.10 to 2.25) | <0.0001 | 2.42 | (2.19 to 2.66) | <0.0001 | 3.17 | (2.33 to 4.32) | <0.0001 |
| 55 to 59 | 2.38 | (2.29 to 2.46) | <0.0001 | 2.64 | (2.40 to 2.91) | <0.0001 | 3.46 | (2.54 to 4.71) | <0.0001 |
| 60 to 64 | 2.66 | (2.57 to 2.76) | <0.0001 | 2.95 | (2.67 to 3.25) | <0.0001 | 3.83 | (2.81 to 5.21) | <0.0001 |
| 65 to 69 | 2.94 | (2.83 to 3.05) | <0.0001 | 3.16 | (2.86 to 3.49) | <0.0001 | 4.18 | (3.07 to 5.69) | <0.0001 |
| 70 to 74 | 3.11 | (2.99 to 2.34) | <0.0001 | 3.38 | (3.06 to 3.73) | <0.0001 | 4.62 | (3.39 to 6.29) | <0.0001 |
| 75 to 79 | 3.42 | (3.28 to 3.57) | <0.0001 | 3.74 | (3.38 to 4.14) | <0.0001 | 5.14 | (3.77 to 7.01) | <0.0001 |
| ≥ 80 | 3.69 | (3.53 to 3.85) | <0.0001 | 4.29 | (3.88 to 4.74) | <0.0001 | 6.09 | (4.47 to 8.30) | <0.0001 |
| **Sex** |  |  |  |  |  |  |  |  |  |
| Male | Reference | - | - | Reference | - | - | Reference | - | - |
| Female | 1.00 | (0.99 to 1.01) | 0.68 | 0.96 | (0.95 to 0.98) | <0.0001 | 0.90 | (0.88 to 0.92) | <0.0001 |
| **Residence** |  |  |  |  |  |  |  |  |  |
| Rural | Reference | - | - | Reference | - | - | Reference | - | - |
| Urban | 1.06 | (1.05 to 1.08) | <0.0001 | 1.05 | (1.02 to 1.08) | 0.0003 | 1.01 | (0.97 to 1.05) | 0.69 |
| **Neighborhood income quintile** |  |  |  |  |  |  |  |  |  |
| Quintile 1 (lowest income) | Reference | - | - | Reference | - | - | Reference | - | - |
| Quintile 2 | 0.96 | (0.95 to 0.98) | 0.0001 | 0.95 | (0.93 to 0.98) | 0.0003 | 0.96 | (0.92 to 0.99 | 0.02 |
| Quintile 3 | 0.94 | (0.92 to 0.96) | <0.0001 | 0.94 | (0.91 to 0.96) | <0.0001 | 0.94 | (0.90 to 0.98) | 0.002 |
| Quintile 4 | 0.92 | (0.90 to 0.94) | <0.0001 | 0.93 | (0.90 to 0.95) | <0.0001 | 0.94 | (0.90 to 0.98) | 0.002 |
| Quintile 5 (highest income) | 0.91 | (0.89 to 0.93) | <0.0001 | 0.90 | (0.88 to 0.93) | <0.0001 | 0.91 | (0.88 to 0.95) | <0.0001 |
| **Primary care enrolment model** |  |  |  |  |  |  |  |  |  |
| Not-enrolled | Reference | - | - | Reference | - | - | Reference | - | - |
| Family Health Group | 1.11 | (1.09 to 1.13) | <0.0001 | 1.01 | (0.98 to 1.03) | 0.67 | 1.03 | (0.99 to 1.06) | 0.12 |
| Family Health Network or  Organization | 1.03 | (1.00 to 1.05) | 0.04 | 0.93 | (0.90 to 0.96) | <0.0001 | 0.96 | (0.92 to 0.99) | 0.03 |
| Family Health Team | 1.00 | (0.97 to 1.02) | 0.75 | 0.91 | (0.88 to 0.94) | <0.0001 | 0.91 | (0.87 to 0.95) | <0.0001 |
| Other† | 1.11 | (1.07 to 1.16) | <0.0001 | 1.02 | (0.97 to 1.07) | 0.39 | 1.04 | (0.97 to 1.11) | 0.28 |
| **Inpatient general practice visits** | 1.03 | (1.02 to 1.06) | <0.0001 | 1.02 | (1.00 to 1.04) | 0.02 | 1.02 | (1.01 to 1.04) | 0.002 |
| **Inpatient specialist visits** | 1.00 | (0.98 to 1.03) | 0.87 | 1.05 | (1.03 to 1.08) | <0.0001 | 1.02 | (0.99 to 1.05) | 0.18 |
| **Outpatient general practice visits** | 1.10 | (1.10 to 1.11) | <0.0001 | 1.09 | (1.09 to 1.10) | <0.0001 | 1.08 | (1.07 to 1.08) | <0.0001 |
| **Outpatient specialist visits** | 1.11 | (1.10 to 1.12) | <0.0001 | 1.10 | (1.08 to 1.12) | <0.0001 | 1.08 | (1.07 to 1.09) | <0.0001 |

Abbreviations: CI = Confidence Interval; CHR = Cause-Specific Hazard Ratio.

Note: The relationship between continuity and a) Time in days until the diagnosis of the 2^nd^ condition among those with at least 1 condition (n = 166,665), b) Time in days until the diagnosis of the 3^rd^ condition among those with at least 2 conditions (n = 119,520), and c) Time in days until the diagnosis of the 4^th^ condition among those with at least 3 conditions (n = 68,021) was estimated with multivariable cause-specific hazards regression models. The effect estimate of continuity was adjusted for age, sex, income, primary care enrolment model, number of physician visits (inpatient general practice, inpatient specialist, outpatient general practice, outpatient specialist), and place of residence simultaneously.

‡Continuity of care was measured using the Bice-Boxerman Index and categorized as high versus low continuity at the median among all patients at index. Visits to family physicians and specialists in an inpatient or outpatient setting (office, home, long-term care, emergency department, telephone, ‘undefined’) were counted in the calculation of continuity, with less than three visits per year treated as missing.

†Comprehensive Care Model, Community Sponsored Agreement, Community Health Group, Group Health Center, Health Services Organization, Primary Care Network, Rural and Northern Group, South Eastern Area Medical Organization, and St. Joseph’s Health Centre.
