# Supplementary material for: Association between continuity of care and subsequent diagnosis of multimorbidity in Ontario, Canada from 2001–2015: A retrospective cohort study
Source: PLoS One. 2021 Mar 11;16(3):e0245193. doi: 10.1371/journal.pone.0245193 (PMC7951913; doi:10.1371/journal.pone.0245193)
Supplement: S9 Table — (DOCX) [file pone.0245193.s009.docx]

S9 Table. Adjusted cause-specific hazard ratios of time-dependent usual provider of care calculated with all inpatient and outpatient family physician and specialist visits (using ≥ 2 visits annually).

|  | **Condition 1 (N = 166,665) to Condition 2** | | | **Condition 2 (N = 119,520) to Condition 3** | | | **Condition 3 (N = 68,021) to Condition 4** | | |
| --- | --- | --- | --- | --- | --- | --- | --- | --- | --- |
| **Characteristic** | CHR | 95% CI | *p-value* | CHR | 95% CI | *p-value* | CHR | 95% CI | *p-value* |
| **Usual Provider of Care‡** |  |  |  |  |  |  |  |  |  |
| Low (≤ 0.75) | Reference | - | - | Reference | - | - | Reference | - | - |
| High (> 0.75) | 0.90 | (0.89 to 0.92) | <0.0001 | 0.88 | (0.86 to 0.89) | <0.0001 | 0.87 | (0.84 to 0.89) | <0.0001 |
| < 2 visits | 0.64 | (0.63 to 0.65) | <0.0001 | 0.52 | (0.50 to 0.53) | <0.0001 | 0.50 | (0.46 to 0.53) | <0.0001 |
| **Age (years)** |  |  |  |  |  |  |  |  |  |
| 18 - 24 | Reference | - | - | Reference | - | - | Reference | - | - |
| 25 - 29 | 1.10 | (1.06 to 1.14) | <0.0001 | 1.25 | (1.12 to 1.40) | <0.0001 | 1.54 | (1.11 to 2.14) | <0.0001 |
| 30 - 34 | 1.25 | (1.21 to 1.30) | <0.0001 | 1.37 | (1.24 to 1.52) | <0.0001 | 1.59 | (1.16 to 2.18) | <0.0001 |
| 35 - 39 | 1.45 | (1.40 to 1.50) | <0.0001 | 1.63 | (1.48 to 1.80) | <0.0001 | 2.04 | (1.50 to 2.79) | <0.0001 |
| 40 - 44 | 1.62 | (1.56 to 1.68) | <0.0001 | 1.80 | (1.63 to 1.98) | <0.0001 | 2.51 | (1.84 to 3.43) | <0.0001 |
| 45 - 49 | 1.91 | (1.85 to 1.98) | <0.0001 | 2.18 | (2.00 to 2.41) | <0.0001 | 2.82 | (2.07 to 3.84) | <0.0001 |
| 50 - 54 | 2.19 | (2.12 to 2.27) | <0.0001 | 2.44 | (2.22 to 2.69) | <0.0001 | 3.21 | (2.36 to 4.37) | <0.0001 |
| 55 - 59 | 2.41 | (2.33 to 2.50) | <0.0001 | 2.67 | (2.42 to 2.94) | <0.0001 | 3.50 | (2.57 to 4.76) | <0.0001 |
| 60 - 64 | 2.71 | (2.61 to 2.81) | <0.0001 | 2.99 | (2.72 to 3.30) | <0.0001 | 3.88 | (2.85 to 5.29) | <0.0001 |
| 65 - 69 | 3.02 | (2.91 to 3.14) | <0.0001 | 3.25 | (2.95 to 3.59) | <0.0001 | 4.27 | (3.14 to 5.81) | <0.0001 |
| 70 - 74 | 3.23 | (3.11 to 3.36) | <0.0001 | 3.52 | (3.18 to 3.88) | <0.0001 | 4.78 | (3.51 to 6.51) | <0.0001 |
| 75 - 79 | 3.60 | (3.44 to 3.74) | <0.0001 | 3.93 | (3.55 to 4.35) | <0.0001 | 5.38 | (3.95 to 7.33) | <0.0001 |
| ≥ 80 | 3.97 | (3.80 to 4.14) | <0.0001 | 4.60 | (4.16 to 5.08) | <0.0001 | 6.48 | (4.76 to 8.82) | <0.0001 |
| **Sex** |  |  |  |  |  |  |  |  |  |
| Male | Reference | - | - | Reference | - | - | Reference | - | - |
| Female | 1.02 | (1.01 to 1.04) | <0.0001 | 0.98 | (0.96 to 0.99) | 0.006 | 0.91 | (0.88 to 0.93) | <0.0001 |
| **Residence** |  |  |  |  |  |  |  |  |  |
| Rural | Reference | - | - | Reference | - | - | Reference | - | - |
| Urban | 1.07 | (1.05 to 1.09) | <0.0001 | 1.06 | (1.04 to 1.09) | <0.0001 | 1.02 | (0.98 to 1.06) | 0.44 |
| **Neighborhood income quintile** |  |  |  |  |  |  |  |  |  |
| Quintile 1 (lowest income) | Reference | - | - | Reference | - | - | Reference | - | - |
| Quintile 2 | 0.96 | (0.94 to 0.98) | <0.0001 | 0.94 | (0.92 to 0.97) | <0.0001 | 0.95 | (0.91 to 0.99) | 0.01 |
| Quintile 3 | 0.93 | (0.92 to 0.95) | <0.0001 | 0.93 | (0.90 to 0.95) | <0.0001 | 0.93 | (0.90 to 0.97) | 0.0006 |
| Quintile 4 | 0.92 | (0.90 to 0.93) | <0.0001 | 0.92 | (0.89 to 0.94) | <0.0001 | 0.93 | (0.89 to 0.97) | 0.0004 |
| Quintile 5 (highest income) | 0.90 | (0.88 to 0.91) | <0.0001 | 0.88 | (0.86 to 0.91) | <0.0001 | 0.90 | (0.86 to 0.93) | <0.0001 |
| **Primary care enrolment model** |  |  |  |  |  |  |  |  |  |
| Not-enrolled | Reference | - | - | Reference | - | - | Reference | - | - |
| Family Health Group | 1.12 | (1.10 to 1.14) | <0.0001 | 1.02 | (0.99 to 1.04) | 0.23 | 1.03 | (1.00 to 1.07) | 0.09 |
| Family Health Network or Organization | 1.01 | (1.00 to 1.04) | 0.35 | 0.91 | (0.88 to 0.94) | <0.0001 | 0.93 | (0.89 to 0.97) | 0.0012 |
| Family Health Team | 0.98 | (0.95 to 1.01) | 0.14 | 0.89 | (0.86 to 0.92) | <0.0001 | 0.88 | (0.84 to 0.92) | <0.0001 |
| Other† | 1.12 | (1.07 to 1.16) | <0.0001 | 1.03 | (0.98 to 1.08) | 0.32 | 1.04 | (0.97 to 1.11) | 0.29 |

Abbreviations: CI = Confidence Interval; CHR = Cause-Specific Hazard Ratio.

Note: The relationship between continuity and a) Time in days until the diagnosis of the 2^nd^ condition among those with at least 1 condition (N = 166,665), b) Time in days until the diagnosis of the 3^rd^ condition among those with at least 2 conditions (N = 119,520), and c) Time in days until the diagnosis of the 4^th^ condition among those with at least 3 conditions (N = 68,021) was estimated with multivariable cause-specific hazards regression models. The effect estimate of continuity was adjusted for age, sex, neighborhood income, primary care enrolment model, number of physician visits (inpatient primary care, inpatient specialist, outpatient primary care, outpatient specialist), and area of residence simultaneously.

‡Continuity was calculated using the Usual Provider of Care Index and categorized as high versus low continuity at the mean among all patients at index. All visits to the most frequently seen family physician or specialist in an inpatient or outpatient setting (office, home, long-term care, emergency department, telephone, ‘undefined’) were counted in the calculation of continuity.

†Comprehensive Care Model, Community Sponsored Agreement, Community Health Group, Group Health Center, Health Services Organization, Primary Care Group, Primary Care Network, Rural and Northern Group, South Eastern Area Medical Organization, and St. Joseph’s Health Centre.
